# ZMIZ2–DUX4–LGR5 Signaling Axis Maintains Stemness Properties and Facilitates Docetaxel Resistance in Triple‐Negative Breast Cancer

**DOI:** 10.1002/advs.78319

**Published:** 2026-10-22

**Authors:** Xueting Gan, Shulei Sun, Ran Hu, Yuheng Feng, Xinhao Wu, Fangqi Liu, Xuyong Lin, Xiupeng Zhang, Xuezhu Rong, Qiang Han

**Affiliations:** ^1^ Department of Pathology the First Hospital and College of Basic Medical Sciences of China Medical University Shenyang China; ^2^ NHC Key Laboratory of Carcinogenesis and Hunan Key Laboratory of Cancer Metabolism Xiangya School of Basic Medical Sciences Central South University Hunan China; ^3^ Department of Respiratory and Critical Care Medicine Tianjin Medical University General Hospital Tianjin China; ^4^ Department of Pathology Shengjing Hospital of China Medical University Shenyang China; ^5^ Department of Neurosurgery First Hospital of China Medical University Shenyang China; ^6^ Department of Pathology the First Hospital of China Medical University Shenyang China

**Keywords:** cancer stem cell, chemotherapy resistance, DUX4, LGR5, triple‐negative breast cancer, ZMIZ2

## Abstract

Triple‐negative breast cancer (TNBC) is a highly aggressive malignancy with limited targeted treatment options and frequent chemoresistant. Through integrated RNA‐seq analysis of docetaxel‐resistant cell lines and three publicly available docetaxel‐resistant TNBC datasets, we identified zinc finger MIZ‐type containing 2 (*ZMIZ2*) as a critical mediator of chemoresistance in TNBC. ZMIZ2 is markedly upregulated in TNBC tissues and chemoresistant specimens and inversely correlates with clinical chemotherapy response. Functional studies have reveal that ZMIZ2 promotes cancer stemness‐associated properties, malignant progression and docetaxel resistance. Mechanistically, ZMIZ2 directly binds to the transcription factor DUX4 via its MIZ domain and cooperatively activates LGR5 transcription by interacting with BRG1 and BAF57, core components of the SWI/SNF chromatin remodeling complex. This ZMIZ2–DUX4–LGR5 axis sustains tumor cell stemness‐associated properties and promotes tumorigenesis, thereby driving therapeutic resistance. We also identify liquiritin as a ZMIZ2 inhibitor that synergises with docetaxel to effectively enhance tumor regression. Collectively, our findings establish ZMIZ2 as a promising therapeutic target and provide a potential strategy to overcome docetaxel resistance in TNBC.

## Graphical abstract

This study identifies ZMIZ2 as a therapeutic target for docetaxel‐resistant triple‐negative breast cancer, sustaining cancer stemness‐associated properties and docetaxel resistance. ZMIZ2 upregulation enhances DUX4‐mediated LGR5 activation through the SWI/SNF components BRG1 and BAF57. Notably, combining docetaxel with liquiritin, a ZMIZ2 inhibitor, enhances tumor regression, highlighting a novel combination strategy for TNBC therapy.

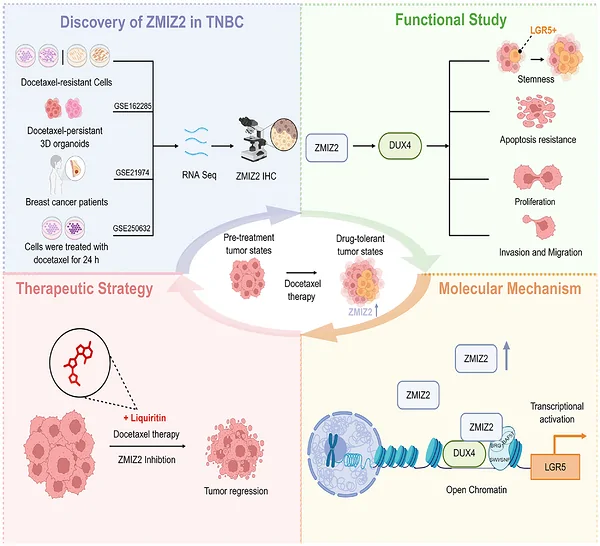

## 1 Introduction

Breast cancer is the most common malignancy in women and remains a major public health challenge worldwide. Triple‐negative breast cancer (TNBC), a subtype comprising 15%–20% of breast cancer cases [1, 2], is characterized by high aggressiveness, rapid progression, and poor prognosis. Although neoadjuvant chemotherapeutic drugs, represented by taxanes (such as docetaxel and paclitaxel) combined with anthracyclines (such as epirubicin and doxorubicin), or platinum‐based drugs, have shown good efficacy in the treatment of TNBC, a substantial proportion of patients develop resistance, resulting in treatment failure [3, 4, 5, 6, 7, 8]. Therefore, deciphering the molecular mechanisms underlying chemoresistance and identifying novel therapeutic targets are urgently needed to improve the prognosis of TNBC patients.

Cancer stem cells (CSCs), characterized by self‐renewal capacity and multipotent differentiation potential, are critically involved in tumor recurrence, metastasis and therapeutic resistance [9]. Indeed, emerging evidence suggests that breast CSCs (BCSCs) contribute to the high recurrence and distant metastasis rates of TNBC and play a central role in drug resistance and immune evasion [10, 11, 12, 13].

Zinc finger MIZ‐type containing 2 (ZMIZ2) is a transcriptional co‐activator that contains a nuclear localization sequence (NLS), an msx‐interacting zinc finger (MIZ) domain, and a C‐terminal proline‐rich region [14]. It has been implicated in the progression of several solid tumors, including prostate, colon, and hepatocellular carcinomas, by enhancing STAT‐dependent transcription and interacting with SWI/SNF chromatin remodeling complex components [14, 15, 16, 17, 18]. In addition, ZMIZ2 has been reported to mediate the oncogenic effects of IGF2BP3/circRARS and SGK3 in renal cell carcinoma and estrogen receptor‐positive (ER+) breast cancer, respectively [19, 20]. More recently, ZMIZ2 has also been shown to promote lung cancer malignancy by regulating the Wnt and Hippo pathways through SIRT1 [21]. However, despite its established roles in multiple malignancies [22], the specific function of ZMIZ2 in TNBC chemoresistance and its underlying molecular mechanisms have not been fully elucidated.

Docetaxel resistance remains a major unmet clinical challenge in TNBC and necessitates the identification of novel therapeutic targets and strategies. Through integrative analyses combining RNA sequencing, bioinformatics, molecular biology, and functional in vitro and in vivo experiments, we identified ZMIZ2 as a critical mediator of TNBC progression and chemoresistance. Notably, we identified liquiritin as a novel targeted small‐molecule inhibitor of ZMIZ2, that enhanced the therapeutic efficacy of docetaxel against ZMIZ2‐high expressing docetaxel‐resistant TNBC cells in preclinical models. These findings establish ZMIZ2 as a promising therapeutic target and offer a novel strategy to overcome docetaxel resistance in TNBC patients who develop treatment failure.

## 2 Results

### 2.1 Identification and Clinical Relevance of ZMIZ2 as a Novel Mediator of Docetaxel Resistance in TNBC

To screen for novel mediators of docetaxel resistance in TNBC, we established two docetaxel‐resistant cell lines, MDA‐MB‐468R and MDA‐MB‐231R through a 6‐month stepwise dose‐escalation protocol. Compared with parental cells, the resistant sublines exhibited significantly elevated IC_50_ values (Figure 1A) and enhanced survival upon docetaxel treatment (Figures 1B,C and S1A,B). Subsequently, we performed RNA‐seq on parental (MDA‐MB‐231 and MDA‐MB‐468) and docetaxel‐resistant (MDA‐MB‐231R and MDA‐MB‐468R) cell lines, revealing 2,463 and 4,295 upregulated genes in MDA‐MB‐231R and MDA‐MB‐468R, respectively (Figure S1C–E). To prioritize clinically relevant candidates, we cross‐referenced our data with three previously published RNA‐seq datasets of docetaxel‐resistant TNBC (GSE21974, GSE162285, and GSE250632) [23, 24, 25, 26]. Notably, *ZMIZ2* emerged as the sole gene consistently upregulated across all four cohorts (Figure 1D). Immunoblotting and reverse transcription‐quantitative polymerase chain reaction (RT‐ qPCR) further corroborated the upregulation of ZMIZ2 at both the protein and mRNA levels in the resistant sublines (Figure 1E).

**FIGURE 1:**
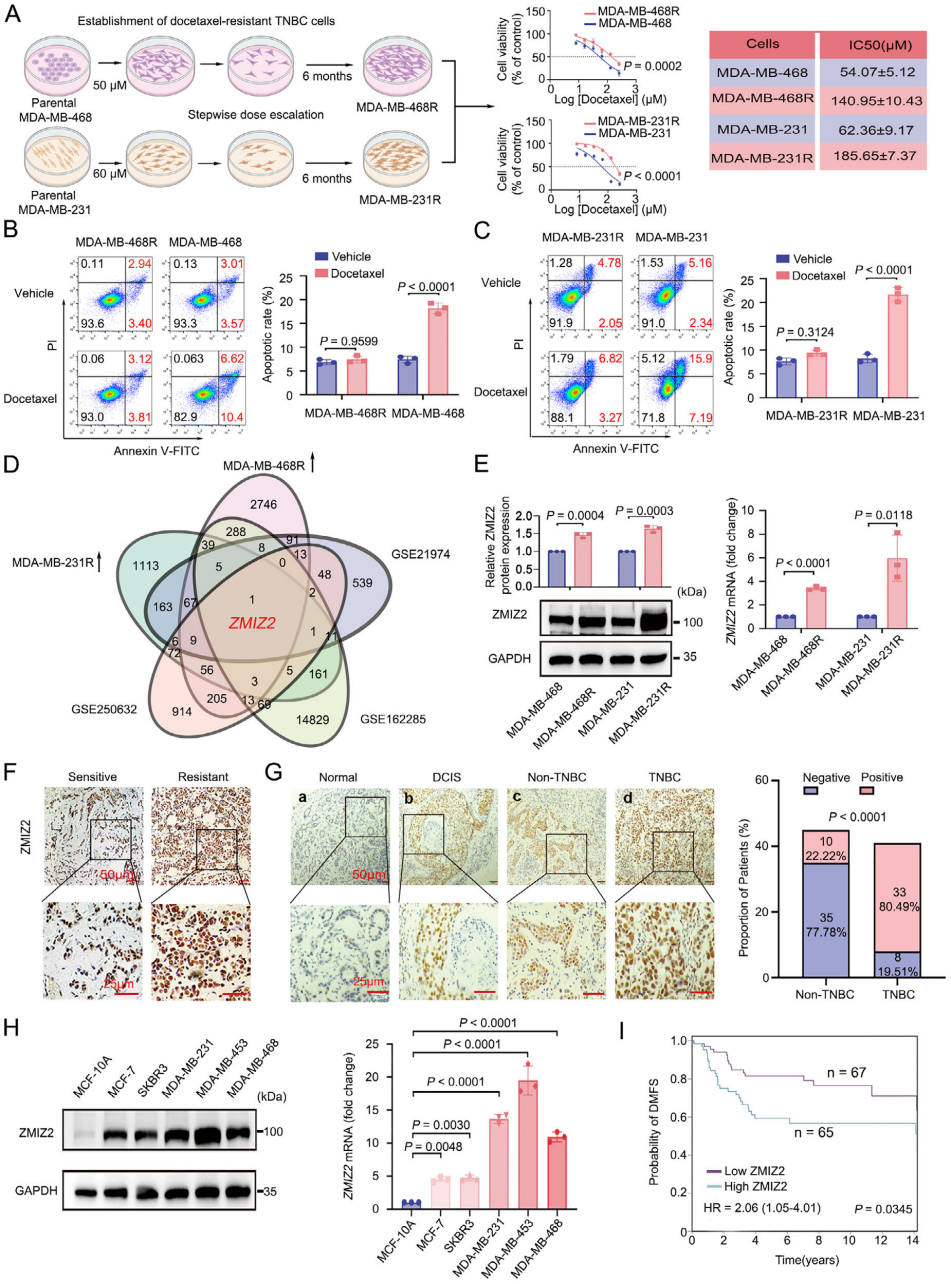
Identification and clinical relevance of ZMIZ2 as a novel mediator of docetaxel resistance in TNBC. (A) Schematic diagram illustrating the stepwise dose‐escalation protocol used to establish docetaxel‐resistant MDA‐MB‐231R and MDA‐MB‐468R cell lines. MTS assays showing the dose‐response curves of parental and docetaxel‐resistant cells after 48 h of docetaxel treatment (8, 16, 32, 64, 128, and 256 µM). The resistant sublines exhibited a rightward shift in the dose‐response curve, with substantially elevated IC_50_ values relative to parental controls (n = 3 independent experiments). (B–C) Flow cytometry analysis of apoptosis rate in resistant and parental cells treated with vehicle or docetaxel (n = 3 independent experiments). (D) Venn diagram showing upregulated genes identified in MDA‐MB‐231R and MDA‐MB‐468R cells, intersected with three Gene Expression Omnibus (GEO) datasets (GSE162285, GSE21974, and GSE250632) for docetaxel‐resistant TNBC models [23, 24, 25, 26]. (E) Immunoblotting and RT‐qPCR confirming the upregulation of ZMIZ2 at the protein and mRNA levels in the resistant sublines compared with parental cells (*n* = 3 independent experiments). (F) Representative IHC micrographs of ZMIZ2 expression in pre‐operative biopsies from 82 TNBC patients receiving docetaxel‐based neoadjuvant chemotherapy, stratified by chemotherapeutic response (Cohort 1, 52 sensitive vs. 30 resistant). See Section 5.3 for IHC scoring details. (G) Representative IHC micrographs of ZMIZ2 expression in 86 post‐mastectomy specimens from treatment‐naïve patients (Cohort 2, 45 non‐TNBC vs. 41 TNBC cases). Representative images showing (a) normal breast ductal epithelium, (b) ductal carcinoma in situ (DCIS), (c) non‐TNBC, and (d) TNBC invasive carcinoma. See Section 5.3 for IHC scoring details. Scale bar: 50 µm (top, 200× magnification) and 25 µm (bottom, 400× magnification). (H) Immunoblotting and RT‐qPCR showing ZMIZ2 expression across a panel of breast cancer cell lines (MDA‐MB‐231, MDA‐MB‐453, and MDA‐MB‐468) compared with normal breast epithelial (MCF‐10A) and non‐TNBC cell lines (MCF7 and SKBR3). GAPDH served as an internal control (*n* = 3 independent experiments). (I) Kaplan–Meier survival curves (derived from www.kmplot.com) showing the relationship between ZMIZ2 expression and distant metastasis‐free survival (DMFS) in 132 patients with TNBC. For panels A–C, E, and H, data are presented as the mean ± standard deviation (SD) from three biologically independent samples. Statistical significance was determined using two‐way analysis of variance (ANOVA) followed by Šídák's multiple comparisons test (A–C), unpaired Student's *t*‐test (E), or one‐way ANOVA followed by Dunnett's multiple comparisons test (H). *P*‐values are indicated in the corresponding panels.

We assessed ZMIZ2 expression in a previously established cohort (Cohort 1) of 82 patients receiving docetaxel‐based neoadjuvant therapy, including 52 drug‐sensitive (Grades III–V) and 30 drug‐resistant (Grades I–II) cases. Immunohistochemistry (IHC) revealed that both the intensity and positivity rate of ZMIZ2 were significantly higher in the non‐pathological complete response (non‐pCR) group than in the pathological complete response (pCR) group (n = 30 and n = 52, respectively). This inverse relationship was further reflected in the strong negative correlation with the Miller–Payne grade (*R* = −0.631, *p* < 0.001) (Figure 1F and Table 1). Extending this analysis to a broader TNBC context, we examined its expression in an independent cohort (Cohort 2) of 86 post‐mastectomy specimens from treatment‐naïve patients (45 non‐TNBC and 41 TNBC cases). ZMIZ2 positivity was enriched in TNBC tissues (80.49%, 33/41) compared with non‐TNBC tissues (22.22%, 10/45; *p* < 0.0001) (Figure 1G). Notably, its expression was positively correlated with advanced pathological tumor‐node‐metastasis (pTNM) stage (*p* = 0.032; Table 2).

**TABLE 1:** Correlation of ZMIZ2 expression with Miller–Payne grade in Cohort 1.

Miller–Payne Grade	ZMIZ2				Spearman *R*‐value	*p*‐value (two‐tail)
	(−)	(+)	(++)	(+++)		
I	0	1	5	10	–0.631	*p* < 0.001
II	0	0	3	11		
III	1	3	7	4		
IV	0	10	5	2		
V	7	8	3	2		
Total	8	22	23	29		

The IHC scoring system is detailed in Section 5.3.

**TABLE 2:** Association of ZMIZ2 expression with clinicopathological features in breast cancer specimens from Cohort 2.

Clinicopathological characteristics	*N*	ZMIZ2‐negative	ZMIZ2‐positive	χ2	*p*‐value (two‐sided)
Age (years)				0.844	0.358
≤45	32	11 (34.38%)	21 (65.63%)		
>45	54	24 (44.44%)	30 (55.56%)		
TNBC				29.132	<0.001
No	45	35 (77.78%)	10 (22.22%)		
Yes	41	8 (19.51%)	33 (80.49%)		
Lymph node metastasis					
Negative	39	15 (38.46%)	24 (61.54%)	0.339	0.561
Positive	47	21 (44.68%)	26 (55.32%)		
TNM classification					
I–II	19	9 (47.37%)	10 (52.63%)	4.591	0.032
III–IV	67	15 (22.39%)	52 (77.61%)		

TNBC, triple‐negative breast cancer. The IHC scoring system is detailed in Section 5.3.

Consistent with these clinical observations, both the protein and mRNA levels of ZMIZ2 were notably upregulated in TNBC cell lines (MDA‐MB‐231, MDA‐MB‐453, and MDA‐MB‐468) compared with normal breast epithelial (MCF‐10A) and non‐TNBC cells (luminal A‐type MCF7 and HER2‐amplified SKBR3) (Figure 1H). Given this elevated expression in TNBC, we examined its prognostic value in breast cancer patients. Kaplan–Meier analysis using a publicly available database [27] revealed that high ZMIZ2 expression (*n* = 65) was associated with shorter distant metastasis‐free survival than low expression (*n* = 67; HR = 2.06 [1.05–4.01], *p* = 0.0345) (Figure 1I). Collectively, these findings identify ZMIZ2 as a critical biomarker associated with docetaxel resistance and malignant progression in TNBC.

### 2.2 ZMIZ2 Enhances the Malignant Phenotype and Confers Docetaxel Resistance in TNBC

To determine whether ZMIZ2 mediates the malignant phenotype and docetaxel resistance in TNBC cells, we generated lentiviral particles overexpressing or knocking down ZMIZ2 expression (via CRISPR‐Cas9) and stably transduced them into both docetaxel‐sensitive and ‐resistant TNBC cell lines (Figures 2A and S2A). Following ZMIZ2 knockdown, the IC_50_ values of MDA‐MB‐231R and MDA‐MB‐468R cells were significantly reduced (Figure 2B). Flow cytometry further revealed that ZMIZ2 knockdown significantly elevated the apoptosis rate of tumor cells in both docetaxel‐treated and untreated groups (Figure 2C). These findings consistently indicate that ZMIZ2 knockdown sensitizes drug‐resistant TNBC cells to docetaxel.

**FIGURE 2:**
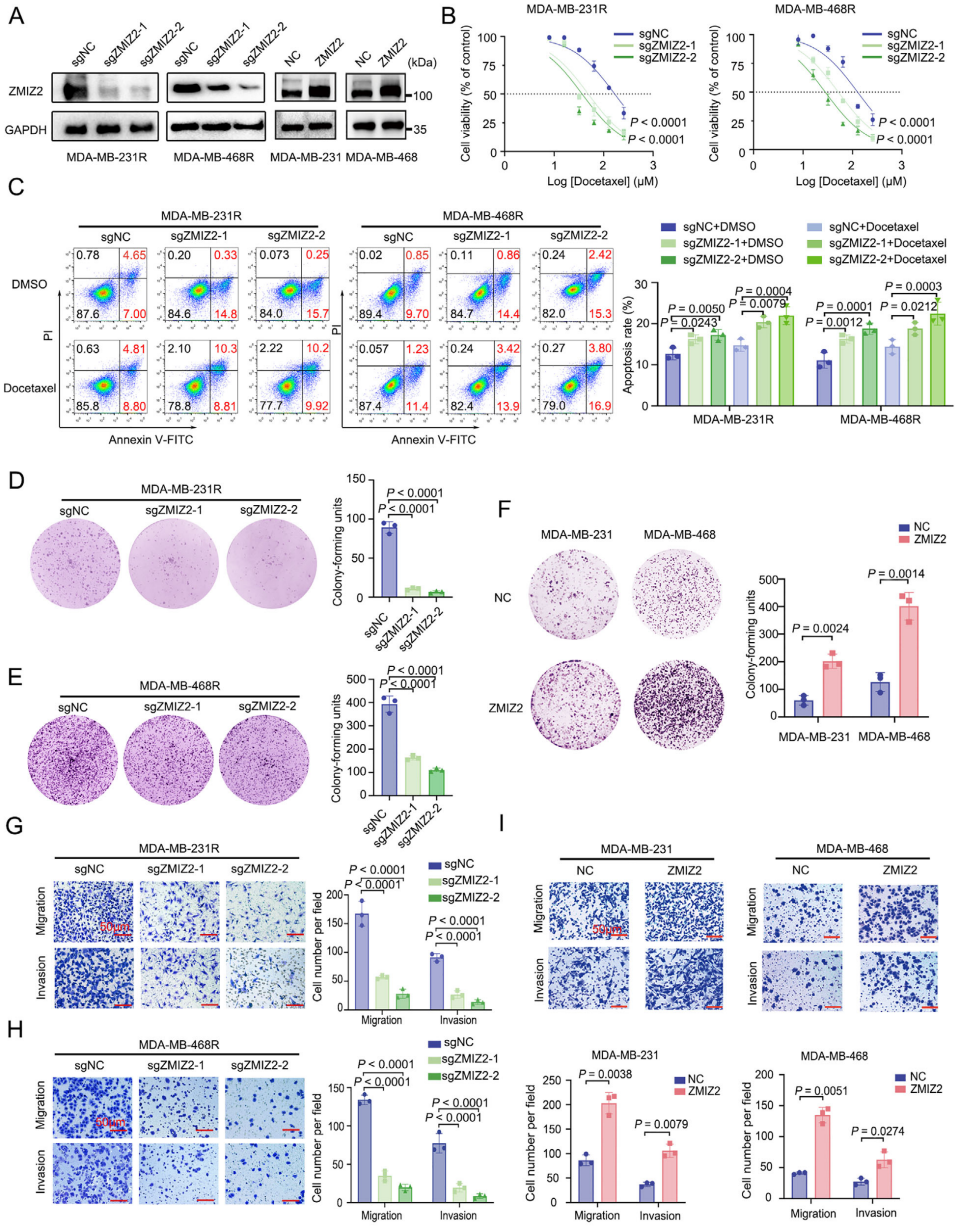
ZMIZ2 enhances the malignant phenotype and confers drug resistance in TNBC. (A) Immunoblotting analysis confirming the efficiency of ZMIZ2 knockdown and overexpression in the indicated TNBC cell lines following lentiviral transduction. (B) MTS assays showing the dose‐response curves of docetaxel‐resistant MDA‐MB‐231 (MDA‐MB‐231R) and MDA‐MB‐468 (MDA‐MB‐468R) cells treated with or without ZMIZ2 knockdown after 48 h of docetaxel treatment (8, 16, 32, 64, 128, and 256 µM). Knockdown of ZMIZ2 shifted the dose‐response curves leftward and significantly reduced the IC_50_ values compared with control cells (sgNC) (*n* = 3 independent experiments). (C) Flow cytometry analysis of apoptosis rates in MDA‐MB‐231R and MDA‐MB‐468R cells transduced with control sgRNA (sgNC) or ZMIZ2 sgRNAs, under DMSO or docetaxel treatment (n = 3 independent experiments). (D–I) In vitro functional assays showing that ZMIZ2 knockdown significantly suppressed colony formation (D–E), migration, and invasion of MDA‐MB‐231R and MDA‐MB‐468R cells (G–H) (*n* = 3 independent experiments). ZMIZ2 overexpression exerted the opposite effects (F, I). Scale bar: 50 µm; magnification: 200×. For panels B–I, data are presented as the mean ± SD from three biologically independent samples. Statistical significance was determined using two‐way analysis of variance (ANOVA) followed by Šídák's multiple comparisons test (B–C) or one‐way ANOVA followed by Dunnett's multiple comparisons test (D–E, G–H). unpaired Student's *t*‐test (F, I). *P*‐values are indicated in the corresponding panels.

We assessed the effect of ZMIZ2 on malignant behaviors. MTS proliferation, colony formation and Transwell assays demonstrated that ZMIZ2 knockdown markedly suppressed cell proliferation (Figure S2B,C), colony formation (Figures 2D,E and S2D,E), migration and invasion, respectively (Figures 2G,H and S2F,G). Conversely, lentivirus‐mediated ZMIZ2 overexpression significantly enhanced these malignant phenotypes (Figure S2H, Figure 2F,I).

For validation, we established an orthotopic breast tumor model to evaluate the therapeutic effects of ZMIZ2 knockdown on docetaxel resistance in TNBC. ZMIZ2 knockdown significantly attenuated tumor growth compared with the control group, regardless of docetaxel treatment, whereas re‐expression of ZMIZ2 restored tumor growth to levels comparable to those of the control group. Notably, in mice receiving docetaxel, ZMIZ2 knockdown resulted in the smallest tumor volumes, lower than those in the untreated control group (Figure S2I–K). Convergently, the in vitro and in vivo findings confirm that ZMIZ2 promotes malignant progression and docetaxel resistance in TNBC.

### 2.3 ZMIZ2 Functions as a Transcriptional Co‐Activator of DUX4 Via Its MIZ Domain

Having established that ZMIZ2 promotes docetaxel resistance and malignant progression in TNBC, we next sought to elucidate the underlying molecular mechanisms. To this end, we analyzed the RNA‐seq data obtained from docetaxel‐resistant TNBC cell lines (MDA‐MB‐231R and MDA‐MB‐468R). A set of differentially expressed genes (DEGs; |log2 fold change| > 0.2, *p* < 0.01) was identified and subjected to Gene Ontology (GO) and Kyoto Encyclopedia of Genes and Genomes (KEGG) pathway enrichment analyses. The results revealed that the DEGs were significantly enriched in transcriptional co‐activator activity and transcription factor binding (Figure 3A,B), consistent with the known function of ZMIZ2. To pinpoint the specific transcription factors co‐activated by ZMIZ2, we intersected our mass spectrometry (MS) data [21] with the JASPAR transcription factor database [28] and the GSE162285 RNA‐seq dataset [25]. This multi‐step intersection analysis identified DUX4 as the only transcription factor that overlapped across all three datasets (Figure 3C). We therefore selected DUX4 for further functional validation.

**FIGURE 3:**
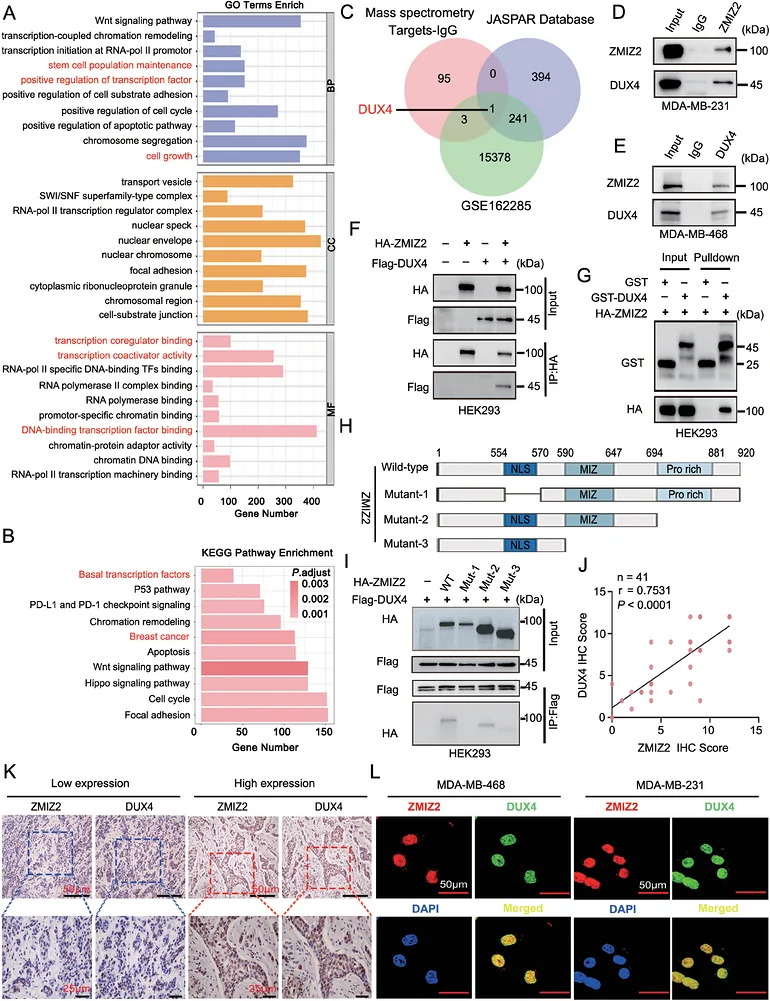
ZMIZ2 functions as a transcriptional co‐activator of DUX4 via its MIZ domain. (A–B) GO and KEGG pathway enrichment analyses of differentially expressed genes in docetaxel‐resistant TNBC cells. (C) Venn diagram showing the intersection of ZMIZ2‐binding proteins identified by MS with transcription factors from the JASPAR database (https://jaspar.genereg.net/) and upregulated genes from the GSE162285 dataset. (D–E) Endogenous interaction between ZMIZ2 and DUX4, as determined by co‐IP assays in MDA‐MB‐231 and MDA‐MB‐468 cells using anti‐ZMIZ2, anti‐DUX4 or control IgG antibodies. (F) Exogenous interaction between HA‐ZMIZ2 and Flag‐DUX4 in HEK293 cells, validated by co‐IP assays. (G) Direct interaction between HA‐ZMIZ2 and GST‐DUX4, as determined by GST pull‐down assay. (H) Schematic diagram illustrating the design of ZMIZ2 truncation mutants, including ZMIZ2‐ΔNLS (mutant‐1), ZMIZ2‐Δ694–920aa (mutant‐2, lacking the proline‐rich region), and ZMIZ2‐Δ590–920aa (mutant‐3, lacking both the MIZ domain and the proline‐rich region). (I) Co‐IP assays mapping the ZMIZ2 domain responsible for DUX4 binding in HEK293 cells co‐transfected with Flag‐DUX4 and HA‐tagged ZMIZ2 truncation mutants. (J–K) Representative IHC micrographs of ZMIZ2 and DUX4 expression in 41 treatment‐naïve TNBC tissue specimens from Cohort 2. See Section 5.3 for IHC scoring details. Scale bar: 50 µm (top, 200× magnification) and 25 µm (bottom, 400× magnification). Spearman's correlation analysis revealed a strong positive correlation between ZMIZ2 and DUX4 levels (*R* = 0.7531, *p* < 0.0001). (L) Immunofluorescence micrographs showing the colocalization of ZMIZ2 and DUX4 in the nuclei of MDA‐MB‐231 and MDA‐MB‐468 cells (scale bar: 25 µm; magnification: 400×).

To assess whether ZMIZ2 and DUX4 can form a stable physical complex, we conducted molecular docking using the HDOCK server. The top‐ranked model (Model_1) predicted a compact interaction interface involving multiple residues (Figure S3A,B) [29, 30, 31, 32, 33, 34]. PRODIGY [35, 36, 37] based binding affinity calculation further supported this prediction, yielding a ΔG of ‐12.2 kcal/mol (K_d_ = 1.0 × 10^−^^9^ M) (Figure S3C).

These computational findings prompted us to experimentally validate the biological interaction between ZMIZ2 and DUX4 using endogenous co‐immunoprecipitation (IP) assays in MDA‐MB‐231 and MDA‐MB‐468 cells (Figure 3D,E). This interaction was recapitulated at the exogenous level by co‐transfecting HA‐ZMIZ2 and Flag‐DUX4 into HEK293 cells (Figure 3F). GST pull‐down assays further demonstrated a direct physical association between the two proteins, independent of other cellular factors (Figure 3G).

To delineate the critical domains mediating this interaction, we designed a series of ZMIZ2 truncation mutants (Figure 3H): ZMIZ2‐ΔNLS (mutant‐1), ZMIZ2‐Δ694–920aa (mutant‐2, lacking the proline‐rich region), and ZMIZ2‐Δ590–920aa (mutant‐3, lacking both the MIZ domain and proline‐rich region). Co‐IP revealed that mutant‐1, which lacks the NLS and is thus unable to enter the nucleus, failed to bind DUX4. Notably, mutant‐2 retained the ability to bind DUX4, whereas mutant‐3 showed almost no binding, indicating that the interaction primarily depends on the MIZ domain of ZMIZ2 (Figure 3I).

We examined the clinical relevance of this interaction in 41 treatment‐naïve TNBC tissue specimens from Cohort 2. A strong positive Spearman's correlation was observed between ZMIZ2 and DUX4 protein levels (*R* = 0.7531, *p* < 0.0001) (Figure 3J,K).

Immunofluorescence assays further demonstrated their co‐localization within the nuclei of TNBC cells (Figure 3L). Collectively, these findings are consistent with a model in which ZMIZ2 directly binds to and co‐activates the transcription factor DUX4 via its MIZ domain in TNBC.

### 2.4 DUX4 Promotes Malignant Progression and Docetaxel Resistance in TNBC

DUX4 is a double homeobox transcription factor with established roles in early embryonic development [38, 39] and the pathogenesis of several diseases, including viral infections [40, 41, 42], facioscapulohumeral muscular dystrophy [43], acute lymphoblastic leukemia [44, 45, 46], undifferentiated small round cell sarcoma [47], and colon cancer [48]. Its potential involvement in breast cancer progression and drug resistance remains largely unexplored.

To address this gap, we first examined DUX4 expression using IHC in 86 treatment‐naïve breast cancer tissue specimens from Cohort 2 (45 non‐TNBC and 41 TNBC cases). The positive rate and staining intensity of DUX4 were significantly higher in TNBC tissues (73.17%, 30/41) than in non‐TNBC tissues (15.56%, 7/45; *p* < 0.0001) (Figure 4A,B). We investigated the functional role of DUX4 in TNBC by stably overexpressing DUX4 in MDA‐MB‐231 and MDA‐MB‐468 cells using lentiviral particles (Figure 4C). Drug sensitivity assays revealed that DUX4 overexpression conferred docetaxel resistance, as evidenced by substantially elevated IC_50_ values in both cell lines (Figure 4D–F).

**FIGURE 4:**
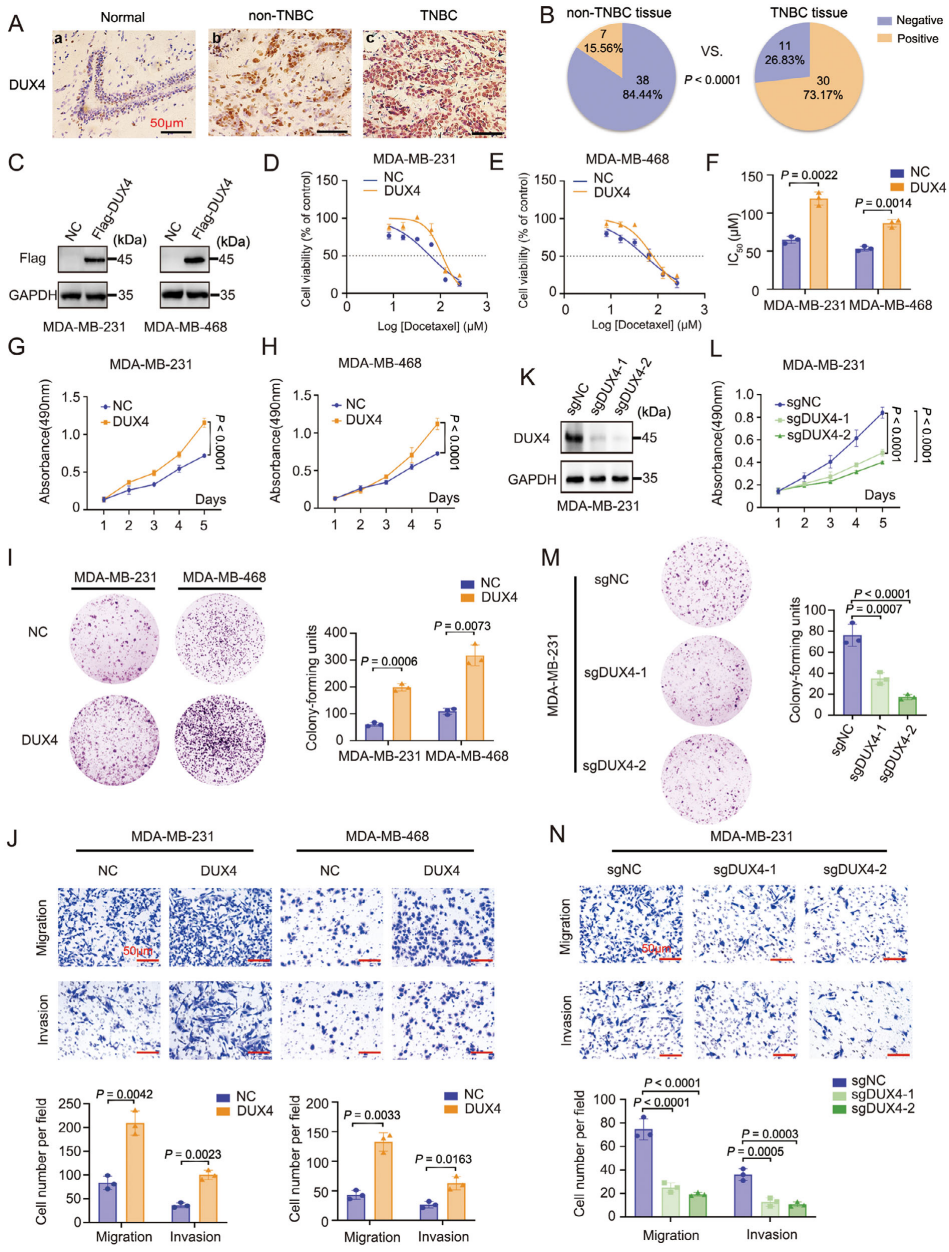
DUX4 promotes malignant progression and docetaxel resistance in TNBC. (A–B) Representative IHC micrographs of DUX4 expression in 86 post‐mastectomy specimens from treatment‐naïve patients (Cohort 2, 45 non‐TNBC vs. 41 TNBC cases). Representative images showing (a) normal breast ductal epithelium, (b) non‐TNBC, and (c) TNBC invasive carcinoma. See Section 5.3 for IHC scoring details. Scale bar: 50 µm; 200× magnification. (C) Immunoblotting analysis confirming the efficiency of DUX4 overexpression in the indicated TNBC cell lines. (D–F) MTS assays showing the dose‐response curves of MDA‐MB‐231 and MDA‐MB‐468 cells with or without lentiviral DUX4 overexpression after 48 h of docetaxel treatment (8, 16, 32, 64, 128, and 256 µM). Overexpressing DUX4 shifted the dose‐response curves rightward and significantly elevated the IC_50_ values compared with control cells (NC) (*n* = 3 independent experiments). (G–J) In vitro functional assays showing that DUX4 overexpression significantly enhanced cell proliferation (G–H), colony formation (I), and migration, and invasion (J) in MDA‐MB‐231 and MDA‐MB‐468 cells (*n* = 3 independent experiments). Scale bar: 50 µm; magnification: 200×. (K–N) Conversely, DUX4 knockdown significantly suppressed cell proliferation (L), colony formation (M), and migration and invasion (N) in MDA‐MB‐231 cells (*n* = 3 independent experiments). Scale bar: 50 µm; magnification: 200×. For panels D–N, data are presented as the mean ± SD from three biologically independent samples. Statistical significance was determined using two‐way ANOVA followed by Šídák's multiple comparisons test (G–H, L), unpaired Student's *t*‐test (D–F, I–J), or one‐way ANOVA followed by Dunnett's multiple comparisons test (M–N). *P*‐values are indicated in the corresponding panels.

We assessed the effect of DUX4 on malignant phenotypes. MTS proliferation, colony formation, and Transwell assays demonstrated that DUX4 overexpression significantly enhanced cell proliferation (Figure 4G,H), colony formation (Figure 4I), and migration and invasion (Figure 4J). This pro‐malignant phenotype was reversed upon DUX4 knockdown in MDA‐MB‐231 cells (Figure 4K), which significantly suppressed these malignant behaviors (Figure 4L–N). Collectively, these gain‐ and loss‐of‐function experiments validate that DUX4 promotes malignant progression and docetaxel resistance in TNBC.

### 2.5 ZMIZ2 Cooperates With BRG1 and BAF57 to Facilitate DUX4‐Mediated LGR5 Transcription

To identify the downstream effectors of the ZMIZ2–DUX4 complex, we performed RNA‐seq on MDA‐MB‐231 cells overexpressing ZMIZ2 or DUX4, identifying 15 and 281 significantly upregulated genes, respectively (|log_2_ fold change| > 1.5, *p* < 0.01). We also performed chromatin IP (ChIP)‐seq to map the genome‐wide binding sites of DUX4, which yielded 4,604 candidate target genes. Intersection of these datasets identified four co‐regulated target genes: *LGR5*, *KITLG*, *PCDH10*, and *TMEM232*. Among them, the CSC‐related *LGR5* ranked as the most significantly upregulated gene (Figure 5A,B).

**FIGURE 5:**
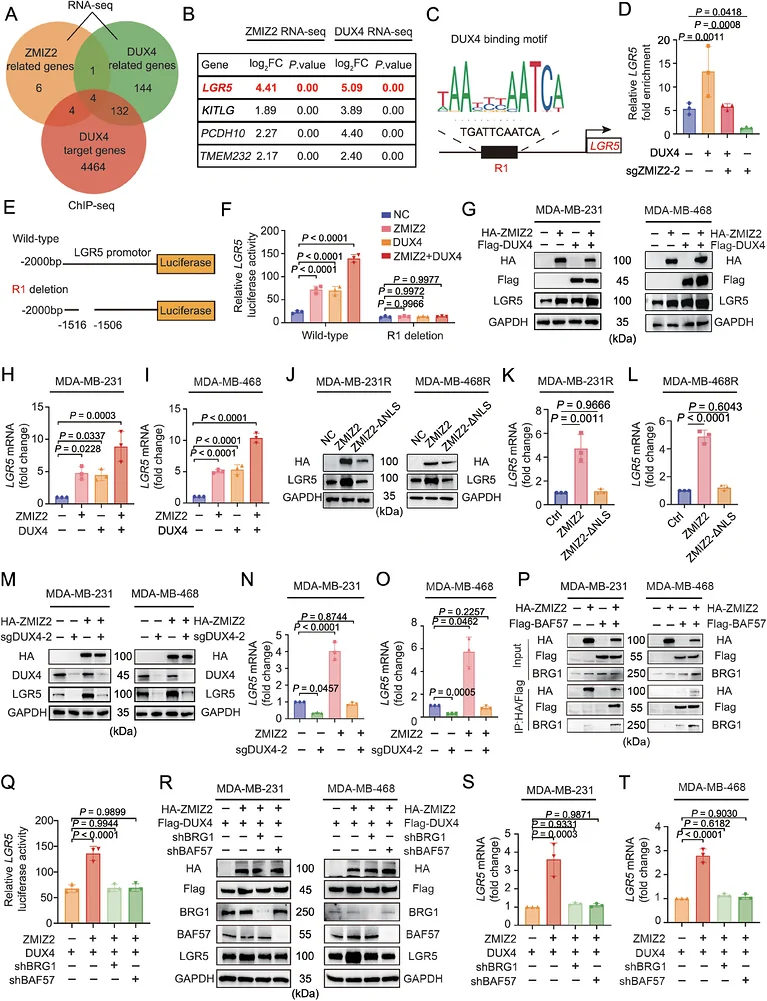
ZMIZ2 cooperates with BRG1 and BAF57 to facilitate DUX4‐mediated LGR5 transcription. (A–B) Venn diagram showing the intersection of RNA‐seq and ChIP‐seq data, identifying four genes co‐regulated by ZMIZ2 and DUX4. (C) Predicted DUX4 binding site in the LGR5 promoter region, as identified by the JASPAR database (https://jaspar.genereg.net/). (D) ChIP‐qPCR analysis confirming the binding of DUX4 to the LGR5 promoter in MDA‐MB‐231 cells, which is dependent on ZMIZ2. (E) Schematic of wild‐type and DUX4 binding site‐deleted LGR5 promoter luciferase reporter constructs. (F) Dual‐luciferase reporter assays showing the synergistic activation of the wild‐type LGR5 promoter by ZMIZ2 and DUX4, with no effect on the DUX4 binding site‐deleted mutant promoter. Renilla luciferase was used for normalization. (G–I) Immunoblotting (G) and RT‐qPCR (H–I) analyses showing that ZMIZ2 and DUX4 independently and synergistically upregulated LGR5 expression in MDA‐MB‐231 and MDA‐MB‐468 cells. (J–L) Immunoblotting (J) and RT‐qPCR (K–L) showing that wild‐type ZMIZ2, but not the NLS‐deficient mutant (ZMIZ2‐ΔNLS), upregulated LGR5 expression. (M–O) Immunoblotting (M) and RT‐qPCR (N–O) showing that ZMIZ2‐mediated upregulation of LGR5 was abolished upon DUX4 knockdown. (P) Co‐IP assays confirming the physical interaction between ZMIZ2 and BRG1/BAF57 in MDA‐MB‐231 and MDA‐MB‐468 cells. (Q) Dual‐luciferase reporter assays showing that knockdown of BRG1 or BAF57 attenuated LGR5 promoter activation by ZMIZ2 and DUX4. Renilla luciferase was used for normalization. (R–T) Immunoblotting (R) and RT‐qPCR (S–T) showing that BRG1 or BAF57 knockdown abolished ZMIZ2–DUX4‐induced upregulation of LGR5 expression. For panels D, F, H–I, K–L, N–O, and Q, S–T data are presented as the mean ± SD from three biologically independent samples. Statistical significance was determined using one‐way ANOVA followed by Tukey's (D) or Dunnett's multiple comparisons test (H–I, K–L, N–O and Q, S–T), or two‐way ANOVA followed by Šídák's multiple comparisons test (F). *P*‐values are indicated in the corresponding panels.

Given the established link between CSCs and TNBC aggressiveness [49], we selected LGR5 for further validation. Sequence analysis of the LGR5 promoter region predicted a potential DUX4 binding site (JASPAR database [50]), which was subsequently confirmed by ChIP‐qPCR assays. Notably, ZMIZ2 knockdown significantly reduced DUX4 occupancy at the LGR5 promoter, indicating that this binding is dependent on ZMIZ2 (Figure 5C,D). Dual‐luciferase reporter assays demonstrated that co‐transfection of ZMIZ2 and DUX4 synergistically enhanced the activity of the wild‐type LGR5 reporter compared with either factor alone, whereas this effect was abolished in both the DUX4 binding‐site deletion mutant and the core‐substitution mutant (Figures 5E,F and S4A,B). Immunoblotting and RT‐qPCR further revealed that ZMIZ2 promoted DUX4‐mediated upregulation of LGR5 in a synergistic manner, while ZMIZ2 alone had a moderate but significant effect (Figure 5G–I). Furthermore, transduction of wild‐type ZMIZ2 upregulated LGR5 expression in docetaxel‐resistant TNBC cells, whereas the NLS‐deficient mutant (ZMIZ2‐ΔNLS), which failed to bind DUX4, had no such effect (Figure 5J–L). Importantly, this ZMIZ2‐mediated upregulation was abolished upon DUX4 knockdown, confirming that ZMIZ2 promotes LGR5 transcription in a DUX4‐dependent manner (Figure 5M–O).

We investigated the molecular mechanism through which ZMIZ2 potentiates DUX4‐mediated LGR5 transcription. Chromatin remodeling is a critical step in the access of transcription factors to promoter regions. ZMIZ2 has been shown to interact with BRG1 (SMARCA4) and BAF57 (SMARCE1), two core components of the SWI/SNF chromatin remodeling complex [14]. Similarly, MS data and exogenous co‐IP assays confirmed that ZMIZ2 physically associates with BRG1 and BAF57 (Figure 5P). We next co‐transfected HA‐ZMIZ2, Flag‐DUX4, and His‐BRG1, or GFP‐BAF57 into HEK293 cells, either alone or in combination, and performed co‐IP assays to confirm the formation of the ZMIZ2–DUX4–BRG1–BAF57 complex. (Figure S4C)

We examined the effect of BRG1 or BAF57 knockdown on LGR5 promoter activity. Luciferase reporter assays showed that knockdown of either significantly attenuated the transcriptional activation of LGR5 by the ZMIZ2–DUX4 complex (Figure 5Q). Immunoblotting and RT‐qPCR further corroborated that BRG1 or BAF57 depletion abrogated the ZMIZ2–DUX4‐induced upregulation of LGR5 (Figure 5R–T). These findings demonstrate that ZMIZ2 cooperates with BRG1 and BAF57 of the SWI/SNF complex to facilitate DUX4‐mediated transcriptional activation of LGR5.

### 2.6 ZMIZ2–DUX4–LGR5 Axis Enhances Stemness‐Associated Properties and Tumorigenicity in TNBC

Given that LGR5 is a well‐established marker of CSCs, we evaluated the regulatory role of ZMIZ2 in TNBC stemness. We examined the effect of ZMIZ2 and DUX4 on the expression of canonical CSC markers, *CD133* and *CD44*, using RT‐qPCR. In both MDA‐MB‐231 and MDA‐MB‐468 cells, overexpression of either ZMIZ2 or DUX4 significantly upregulated *CD133* and *CD44* mRNA levels, and co‐transduction of both further enhanced this effect (Figures 6A and S5A). Conversely, DUX4 knockdown abrogated the ZMIZ2‐mediated upregulation of these markers (Figures 6B and S5B).

**FIGURE 6:**
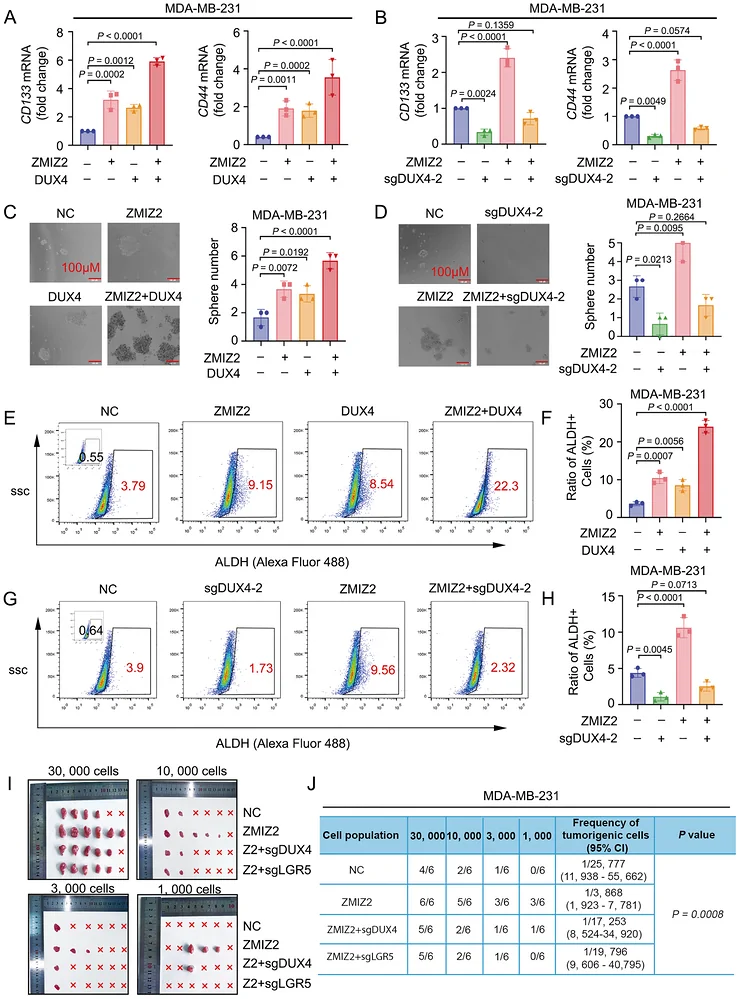
ZMIZ2–DUX4–LGR5 axis enhances stemness‐associated properties and tumorigenicity in TNBC. (A–B) RT‐qPCR of *CD133* and *CD44* mRNA expression in MDA‐MB‐231 cells co‐transduced with ZMIZ2 and DUX4 or with ZMIZ2 and sgDUX4 (*n* = 3 independent experiments). (C–D) Sphere formation assays showing that co‐transduction of ZMIZ2 and DUX4 enhanced sphere‐forming capacity (C), whereas DUX4 knockdown reversed this effect (D) (*n* = 3 independent experiments). Scale bar: 100 µm; magnification,: 100×. (E–H) ALDEFLUOR flow cytometry showing that ZMIZ2 and DUX4 co‐overexpression significantly increased the percentage of ALDH+ cells (E–F), and this effect was reversed upon DUX4 knockdown (G–H) (*n* = 3 independent experiments). (I–J) Limiting dilution xenograft tumor assays in nude mice showing that ZMIZ2 overexpression enhanced tumorigenicity, whereas DUX4 or LGR5 knockdown diminished this effect (*n* = 6 mice per group). For panels A–H, data are presented as the mean ± SD from three biologically independent samples. Statistical significance was determined using one‐way ANOVA, followed by Dunnett's multiple comparisons test (A–H). *P*‐values are indicated in the corresponding panels.

We assessed the effect of the ZMIZ2–DUX4 axis on CSC functional properties. Sphere formation assays demonstrated that cells co‐transduced with ZMIZ2 and DUX4 exhibited significantly enhanced sphere‐forming capacity compared with those transduced with either factor alone (Figures 6C and S5C), whereas DUX4 knockdown eliminated this effect (Figures 6D and S5D). Consistent with these observations, ALDEFLUOR flow cytometry assays revealed that the percentage of ALDH+ tumor stem cells was markedly increased in the co‐transduction group relative to single‐transduction groups (Figures 6E,F and S5E), and this increase was completely reversed upon DUX4 knockdown (Figures 6G,H and S5F).

To validate the in vivo relevance of these findings, we performed limiting dilution xenograft tumor experiments in nude mice. ZMIZ2 overexpression significantly enhanced tumorigenicity, whereas DUX4 or LGR5 knockdown diminished this effect (Figure 6I–J). Together, these findings establish that the ZMIZ2–DUX4–LGR5 axis promotes TNBC stemness‐associated properties and tumorigenicity.

### 2.7 ZMIZ2 Promotes Drug Resistance and the Malignant Phenotype in TNBC via DUX4

Given that CSCs are a major cause of chemoresistance [51, 52], we evaluated the cooperative effects of ZMIZ2 and DUX4 on docetaxel sensitivity and malignant progression in TNBC. Drug sensitivity assays revealed that the IC_50_ value of docetaxel was significantly higher in cells co‐overexpressing ZMIZ2 and DUX4 than in those overexpressing either factor alone (Figures 7A and S6A). Conversely, DUX4 knockdown significantly reduced the IC_50_ value in ZMIZ2‐overexpressing cells (Figures 7B and S6B). Flow cytometry showed that ZMIZ2 overexpression significantly decreased the apoptosis rate of tumor cells with or without docetaxel treatment, whereas DUX4 knockdown restored the apoptosis rate to levels comparable to those of the control group (Figures 7C,D and S6C,D).

**FIGURE 7:**
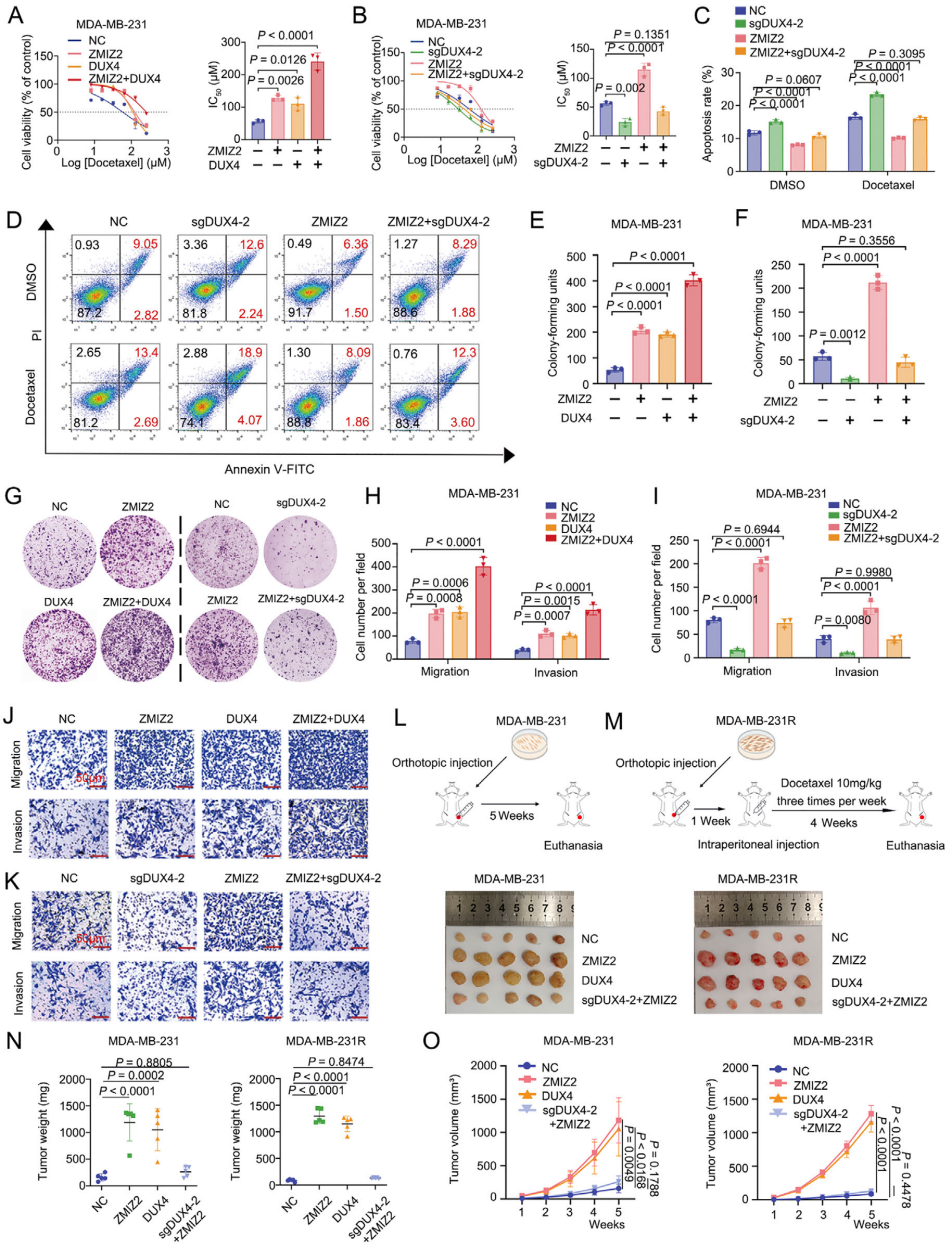
ZMIZ2 promotes drug resistance and the malignant phenotype in TNBC via DUX4. (A–B) MTS assays showing the dose‐response curves of MDA‐MB‐231 cells co‐transduced with ZMIZ2 and DUX4 or with ZMIZ2 and DUX4 sgRNA after 48 h of docetaxel treatment (8, 16, 32, 64, 128, and 256 µM). Overexpression of both ZMIZ2 and DUX4 shifted the dose‐response curves rightward and significantly elevated the IC_50_ values compared with those in control cells (NC)(A). Conversely, DUX4 knockdown shifted the dose‐response curves leftward and significantly reduced the IC_50_ value in ZMIZ2‐overexpressing cells (B) (*n* = 3 independent experiments). (C–D) Flow cytometry of apoptosis rates in MDA‐MB‐231 cells co‐transduced with ZMIZ2 and DUX4 sgRNA under DMSO or docetaxel treatment (*n* = 3 independent experiments). (E–K) In vitro functional assays showing that co‐overexpression of ZMIZ2 and DUX4 significantly enhanced colony formation (E, G) and migration and invasion (H, J) in MDA‐MB‐231 cells. Conversely, DUX4 knockdown abolished these ZMIZ2‐mediated effects (F–G, I and K) (*n* = 3 independent experiments). Scale bar: 50 µm; magnification: 200×. (L–M) Schematic diagram of orthotopic tumorigenesis model and representative images of mouse orthotopic tumors (*n* = 5 mice per group). (N–O) Tumor weight (N) was measured at the endpoint, and tumor volume (O) was measured weekly throughout the indicated period (*n* = 5 mice per group). For panels A–K, data are presented as the mean ± SD from three biologically independent samples. Statistical significance was determined using two‐way ANOVA followed by Šídák's multiple comparisons test (C–D) or one‐way ANOVA followed by Dunnett's multiple comparisons test (A–B, E–K). For panels N–O, data are presented as the mean ± SD from five mice per group. Statistical significance was determined using one‐way (N) or two‐way (O) ANOVA, followed by Dunnett's multiple comparisons test. *P*‐values are indicated in the corresponding panels.

We assessed the effects of ZMIZ2 and DUX4 on malignant phenotypes. Colony formation and Transwell assays demonstrated that co‐overexpression of ZMIZ2 and DUX4 significantly increased colony formation (Figures 7E,G and S6E) and migration and invasion, respectively (Figures 7H,J and S6G,H) compared with either factor alone. Importantly, DUX4 knockdown abolished these ZMIZ2‐mediated effects (Figures 7F,G,I,K and S6F,I,J).

We next assessed the effect of ZMIZ2 and DUX4 on malignant phenotypes in docetaxel‐‑resistant cells. MDA‑MB‑231R and MDA‑MB‑468R cells were transduced with ZMIZ2 alone, DUX4 alone, or co‑transduced with ZMIZ2+sgDUX4. Colony formation and Transwell assays demonstrated that ZMIZ2 overexpression significantly enhanced colony formation and migration/invasion, whereas DUX4 knockdown abolished these ZMIZ2‑mediated effects (Figure S7A–E).

To validate these findings in vivo, we performed orthotopic tumor formation assays in mice (Figure 7L–M). Tumors derived from MDA‐MB‐231 cells and their drug‐resistant counterparts stably overexpressing ZMIZ2 or DUX4 exhibited enhanced growth compared with control tumors. Conversely, DUX4 knockdown significantly attenuated the tumor‐promoting effect of ZMIZ2 (Figure 7N,O). These findings demonstrate conclusively that ZMIZ2 promotes docetaxel resistance and malignant progression in TNBC through a DUX4‐dependent mechanism.

### 2.8 Liquiritin Sensitizes TNBC to Docetaxel and Suppresses Stemness‐Associated Properties by Targeting ZMIZ2

To identify a small‐molecule inhibitor of ZMIZ2 capable of suppressing TNBC malignancy and chemoresistance, we predicted the crystal structure of ZMIZ2 using AlphaFold2 and processed it using Schrödinger software. Molecular docking analysis revealed that liquiritin (LIQ) exhibited the highest binding stability with ZMIZ2 (Figure 8A,B). This interaction was further validated by surface plasmon resonance (SPR) assays, which confirmed the effective binding affinity of LIQ for ZMIZ2 (K_D_: 9.448 mM) (Figure 8C,D). Additionally, cellular thermal shift assays (CETSA) demonstrated the binding of LIQ to ZMIZ2 in living cells (Figure 8E,F).

**FIGURE 8:**
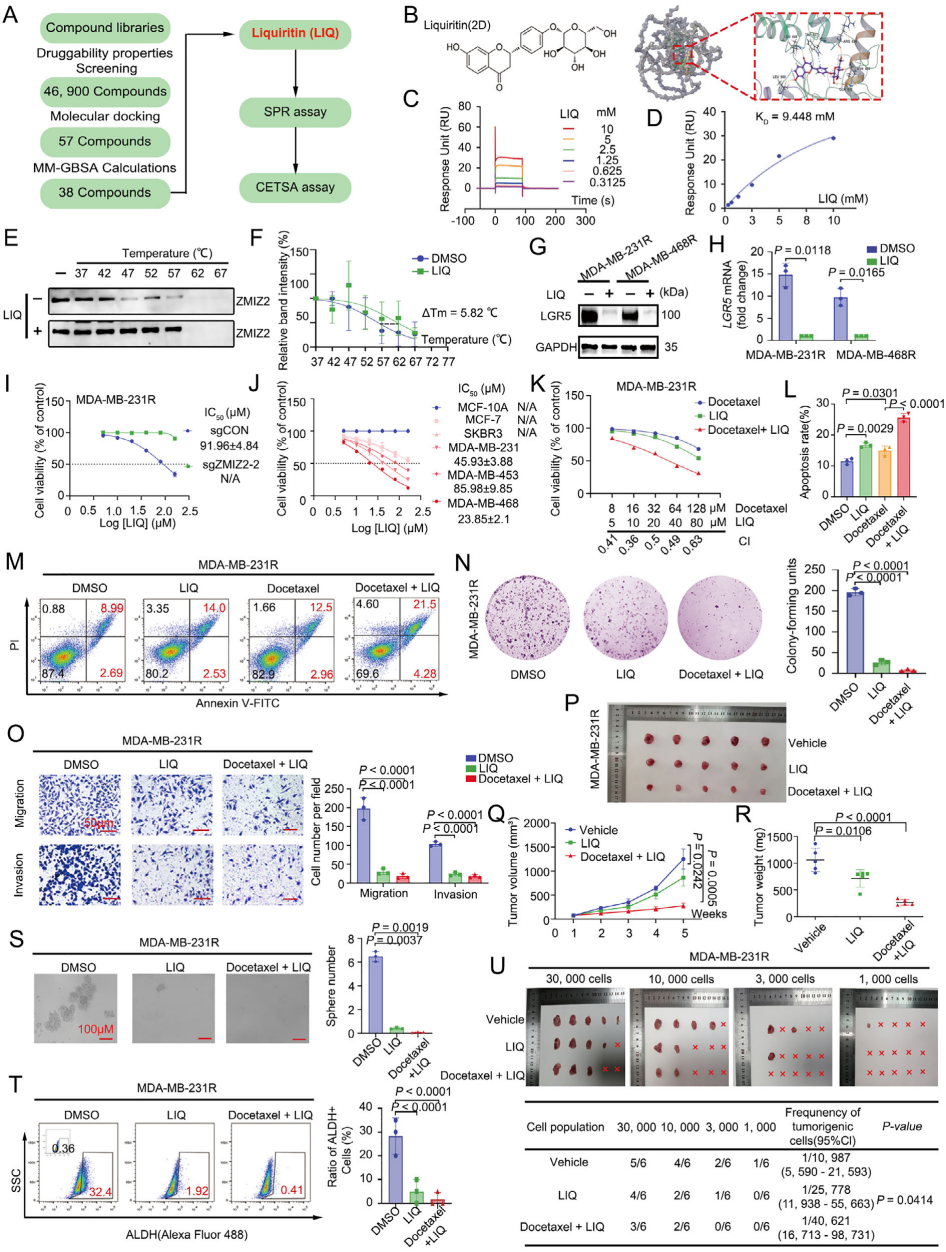
Liquiritin sensitizes TNBC to docetaxel and suppresses stemness‐associated properties by targeting ZMIZ2. (A) Screening strategy used to identify LIQ as a ZMIZ2‐binding compound. (B) Chemical structure of LIQ and its predicted binding mode with ZMIZ2. (C–D) SPR binding curves for LIQ interacting with full‐length ZMIZ2 at the indicated concentrations. (E–F) CETSA showing the thermal stabilization of ZMIZ2 by LIQ (120 µM) in MDA‐MB‐231R cells (E), with the corresponding melt curve and Δ*T_m_* calculated using GraphPad Prism (F) (*n* = 3 independent experiments). (G–H) Immunoblotting (G) and RT‐qPCR (H) of LGR5 protein and mRNA levels in the indicated cell lines treated with or without LIQ (MDA‐MB‐231R: 40 µM; MDA‐MB‐468R: 20 µM) (*n* = 3 independent experiments). (I–J) Drug sensitivity of LIQ in ZMIZ2‐knockdown MDA‐MB‐231R cells (I) and across a panel of cell lines (J). IC_50_ values were estimated using a non‐linear fitting method. (K–M) Effects of LIQ combined with docetaxel on drug‐resistant MDA‐MB‐231R cells, as determined by drug sensitivity (K) and apoptosis assays (L–M). Synergy was calculated using Compusyn software (CI < 1.0). (N–O) Colony formation (N) and Transwell assays (O) showing the synergistic inhibitory effects of LIQ and docetaxel on colony formation, migration, and invasion in MDA‐MB‐231R cells (*n* = 3 independent experiments). Scale bar: 50 µm; magnification: 200×. (P–R) Representative images of subcutaneous tumors from MDA‐MB‐231R cell xenografts in nude mice treated with LIQ and docetaxel (P). Tumor volume (Q) measured weekly throughout the indicated period, and tumor weight (R) recorded at the endpoint (*n* = 5 mice per group). (S–T) Sphere formation assay (S) and ALDEFLUOR flow cytometry (T) showing that LIQ and docetaxel synergistically inhibited sphere formation and reduced the proportion of ALDH+ MDA‐MB‐231R cells (*n* = 3 independent experiments). Scale bar: 100 µm; magnification: 100×. (U) Representative images of transplanted tumors from the limiting dilution tumor propagation assay in nude mice (*n* = 6 mice per group). For panels H, L–O, and S–T, data are presented as the mean ± SD from three biologically independent samples. Statistical significance was determined using unpaired Student's *t*‐test (H) or one‐way ANOVA followed by Tukey's (L–M) or Dunnett's multiple comparisons test (N–O, S–T). For panels Q–R, data are presented as the mean ± SD from five mice per group. Statistical significance was determined using two‐way (Q) or one‐way (R) ANOVA followed by Dunnett's multiple comparisons test. *P*‐values are indicated in the corresponding panels.

We examined whether LIQ functions by inhibiting ZMIZ2 activity. Immunoblotting and RT‑qPCR showed that LIQ significantly reduced LGR5 protein and mRNA levels compared with DMSO treatment, whereas it exerted no regulatory effect on ZMIZ2 or DUX4 expression (Figures 8G,H and S5A). To further validate ZMIZ2 as the functional target of LIQ, we knocked down ZMIZ2 in docetaxel‑resistant MDA‑MB‑231R and MDA‑MB‑468R cells using sgZMIZ2 lentiviral particles. Drug sensitivity assays revealed that ZMIZ2 knockdown markedly reduced the sensitivity of both cell lines to LIQ, with IC_50_ values falling below the detectable range (Figures 8I and S5B). Colony formation and Transwell assays demonstrated that ZMIZ2 knockdown alone did not significantly affect cell proliferation, migration, or invasion, regardless of LIQ treatment (Figure S5C–E). Moreover, sphere formation and ALDEFLUOR assays showed that ZMIZ2 depletion had no apparent impact on stemness properties (Figure S5F–H).

Having established that LIQ acts in a ZMIZ2‑dependent manner, we assessed its specificity for ZMIZ2 using drug sensitivity assays across a panel of cell lines, including normal breast epithelial (MCF‑10A), non‑TNBC (MCF‑7 and SKBR3), and TNBC cells (MDA‑MB‑231, MDA‑MB‑468, and MDA‑MB‑453). Notably, in cells with low or absent ZMIZ2 expression, the IC_50_ values of LIQ fell below the detectable range, indicating minimal impact on cell viability due to the lack of a target. In contrast, in cells with high ZMIZ2 expression, the IC_50_ values of LIQ increased in a roughly dose‑dependent manner parallel to ZMIZ2 expression (Figure 8J). Together, these findings suggest that LIQ selectively inhibits ZMIZ2 activity, rather than altering its expression.

We evaluated the synergistic effects of LIQ with docetaxel. Drug sensitivity assays revealed that LIQ significantly sensitized the drug‐resistant cells to docetaxel (Figures 8K and S6A). Flow cytometry analysis showed that the apoptosis rate was significantly higher in drug‐resistant cells treated with a combination of LIQ and docetaxel than in those treated with docetaxel alone (Figures 8L–M and S6B). Furthermore, colony formation, Transwell, and subcutaneous xenograft assays demonstrated that LIQ synergized with docetaxel to inhibit colony formation, migration, invasion, and transplanted tumor growth (Figures 8N–R and S6C,D). Throughout the in vivo experiments, no significant differences in body weight were observed among the groups, indicating that the combination treatment was well tolerated (Figure S6E,F).

Finally, we assessed the effect of the LIQ–docetaxel combination on CSC properties. Sphere formation and ALDEFLUOR flow cytometry assays revealed that the combination treatment reduced the sphere formation capacity (Figures 8S and S6G) and the percentage of ALDH+ cells (Figures 8T and S6H) compared with the control group. Furthermore, limiting dilution xenograft experiments showed that LIQ–docetaxel reduced tumor incidence in drug‐resistant cells (Figure 8U). In conclusion, LIQ enhances the sensitivity of drug‐resistant TNBC cells to docetaxel by inhibiting ZMIZ2 activity and thereby weakening TNBC stemness‐associated properties.

## 3 Discussion

Through high‐throughput sequencing and comprehensive molecular, cellular, and bioinformatics analyses, this study demonstrated that ZMIZ2 is highly expressed in docetaxel‐resistant TNBC and is significantly associated with poor patient prognosis. This finding is consistent with the established role of ZMIZ2 as an oncoprotein in various malignancies [14, 15, 16, 17, 18, 19, 20, 21, 22]. However, the mechanisms underlying its upregulation in TNBC and chemoresistant TNBC tissues remain unclear. Previous studies have implicated multiple regulatory pathways, including miRNA‐mediated mRNA stabilization (e.g. LINC00265 in colon cancer [17]), m6A‐dependent post‐transcriptional regulation (e.g. IGF2BP3/circRARS complex [19]), and STAT3 phosphorylation‐enhanced transcriptional activation [20]. Additionally, a ZMIZ2:ACTB gene fusion has been identified in adnexal carcinoma [53], suggesting a potential genetic basis for its overexpression in some tumor types. A limitation of this study is the lack of complete follow‐up data for the breast cancer cohort, owing to an insufficient collection period, which prevented a more comprehensive survival analysis.

Since ZMIZ2 primarily functions as a transcriptional co‐activator and promotes malignant progression in various tumors [14, 15, 16, 17, 18, 19, 20, 21, 22], we investigated its potential molecular partners in TNBC. Through mass spectrometry and bioinformatics analyses, we identified DUX4 as a novel transcriptional partner of ZMIZ2. Although DUX4 has been previously associated with cancer progression [44, 45, 46, 47, 48], we found that it is also highly expressed in TNBC, where it functions as an oncoprotein. Mechanistically, DUX4 synergizes with ZMIZ2 to activate transcription at the LGR5 promoter region. LGR5, a well‐established CSC marker similar to CD133, CD44, and ALDH1 [54, 55, 56, 57], is overexpressed in TNBC and has been linked to enhanced invasiveness, epithelial–mesenchymal transition, stem cell enrichment, self‐renewal, and tumorigenicity, as well as recurrence, drug resistance, and poor prognosis [58, 59, 60, 61]. Moreover, we demonstrated that ZMIZ2 acts as a transcriptional co‐activator and cooperates with BRG1 and BAF57, two core components of the SWI/SNF chromatin‐remodeling complex, to facilitate DUX4‐mediated transcriptional activation of LGR5. Although we do not yet have direct evidence that ZMIZ2 facilitates DUX4‐dependent chromatin remodeling at the LGR5 locus, our findings are consistent with this model and provide a strong basis for further investigation. This limitation will be addressed in future studies using chromatin accessibility assays, such as ChIP‐qPCR/CUT&Tag/CUT&RUN. Of note, ZMIZ2 has been reported to enhance the transcriptional activity of androgen receptor (AR), estrogen receptor (ER), thyroid hormone receptor (TR), and vitamin D receptor (VDR) [14], suggesting that it may function as a broad transcriptional co‐activator beyond the ZMIZ2–DUX4–LGR5 axis. In parallel, ZMIZ2 has recently been reported to possess SUMO E3 ligase activity [62]; however, whether ZMIZ2 regulates DUX4 via SUMOylation to enhance its transcriptional activity remains to be determined.

Finally, we systematically elucidated the functional importance of the ZMIZ2–DUX4–LGR5 signaling axis in maintaining TNBC stemness‐associated properties and identified liquiritin (LIQ) as a small‐molecule compound that directly binds to the active site of ZMIZ2. In this context, it is worth noting that the ZMIZ family comprises ZMIZ1 and ZMIZ2, both of which share conserved domains, but exhibit notable differences in their primary structures and active‐site conformations [14]. While our current data—including SPR, CETSA, and ZMIZ2‐knockdown functional assays—strongly support that LIQ specifically targets ZMIZ2, direct binding assays against ZMIZ1 will further confirm this specificity. We plan to address this in our future studies.

Docetaxel‐based regimens remain a cornerstone of TNBC chemotherapy; however, primary or acquired resistance develops in a substantial proportion of patients, leaving limited salvage options. The current management of taxane‐refractory TNBC largely relies on sequential monochemotherapy with agents, such as capecitabine, gemcitabine, or eribulin, while PARP inhibitors benefit only a narrow BRCA‐mutated subset [63, 64, 65, 66, 67]. Although recent advances in Trop‐2–targeted ADCs and immune checkpoint inhibitors have expanded the therapeutic armamentarium for TNBC, durable responses remain elusive for most patients and resistance to these modalities ultimately ensues [68, 69, 70, 71]. In this context, LIQ has been reported to exert antitumor effects in various malignancies, including non‐small cell lung [72], cervical [73], and gastric cancer [74]. One study demonstrates that LIQ combined with cisplatin suppresses tumor growth [75]. In this study, we further demonstrated that LIQ in combination with docetaxel effectively suppresses stemness‐associated properties, chemoresistance, and malignant progression in TNBC. Notably, our findings reveal that LIQ enhances the anti‐tumor efficacy of docetaxel by targeting the ZMIZ2‐DUX4‐LGR5 axis, thereby disrupting CSC maintenance in resistant TNBC cells. This combination strategy provides a novel approach to overcome docetaxel resistance in patients with TNBC who have failed standard chemotherapy. Collectively, these findings provide a robust experimental foundation for the development of novel therapeutic strategies for docetaxel‐resistant TNBC.

## 4 Conclusion

ZMIZ2 is highly expressed in TNBC, particularly in docetaxel‐resistant cases. It functions as a transcriptional co‐activator and interacts with key components of the SWI/SNF chromatin remodeling complex, BRG1 and BAF57, thereby cooperatively enhancing DUX4‐mediated transcriptional activation of LGR5. The ZMIZ2–DUX4–LGR5 signaling axis promotes the stemness‐associated properties of tumor cells, ultimately leading to drug resistance and malignant progression in TNBC. Notably, liquiritin represents a novel ZMIZ2‐targeted small‐molecule agent with promising clinical potential for overcoming docetaxel resistance in TNBC.

## 5 Methods

### 5.1 Cell Culture

All cell lines used in this study were purchased from the Cell Bank of the Chinese Academy of Sciences (Shanghai, China) and were authenticated via short tandem repeat profiling, and confirmed negative for Mycoplasma contamination. MDA‐MB‐231 (#TCHu227) and MDA‐MB‐468 (#TCHu136) cells were cultured in L‐15 medium (#L1518; Sigma‐Aldrich, St. Louis, MO, USA). MCF‐7 (#TCHu74), SK‐BR‐3 (#TCHu225), and MDA‐MB‐453 (#TCHu233) cells were maintained in Dulbecco's modified Eagle's medium (DMEM) (D6429; Sigma‐Aldrich). The human mammary epithelial cell line MCF‐10A (#SCSP‐575) was cultured in Mammary Epithelial Cell Medium (#CM‐0525; Procell, Wuhan, China). All culture media were supplemented with 10% fetal bovine serum (#A5256701; Gibco, Thermo Fisher Scientific, Waltham, MA, USA) and 100 U/mL penicillin–streptomycin (#C0222; Beyotime Biotechnology, Shanghai, China). All cells were cultured at 37°C in a humidified atmosphere containing 5% CO_2_, except for MDA‐MB‐231 and MDA‐MB‐468 cells, which were maintained in a CO_2_‐free environment as recommended by the supplier.

### 5.2 Specimen Collection and Ethics Statements

A total of 168 pathological tissue specimens were collected from the Department of Pathology at the First Hospital of China Medical University (Shenyang, China). Among them, 82 post‐neoadjuvant chemotherapy biopsy specimens from patients with TNBC who received neoadjuvant chemotherapy between 2016 and 2021 constituted Cohort 1. Of these, 58 patients received the EC‐T regimen (four cycles of epirubicin plus cyclophosphamide, followed by four cycles of docetaxel), whereas the remaining 24 patients received the TCbH regimen (six cycles of docetaxel, carboplatin, and trastuzumab). Based on the Miller–Payne grading system, 30 patients were classified as grades I–II, and the other 52 patients were classified as grades III–V.

In addition, 86 treatment‐naïve post‐surgical resection specimens were obtained from patients with breast cancer (Cohort 2), including 41 patients with TNBC and 45 patients with non‐TNBC tumors. All patients underwent complete surgical resection at the Affiliated Cancer Hospital of China Medical University between 2001 and 2003. None of the patients received radiotherapy or chemotherapy prior to surgery, and all received routine adjuvant chemotherapy postoperatively. All patients provided informed consent, and the study was approved by the Ethics Committee of China Medical University (No. LS [2025]003).

### 5.3 Immunohistochemistry

Tissue sections (4 µm thick) were deparaffinized in xylene and rehydrated through a graded ethanol series. Antigen retrieval was performed by boiling in citrate buffer (0.01 M, pH 6.0) for 2 min. After rinsing with phosphate‐buffered saline (PBS, pH 7.5), the sections were immersed in 3% hydrogen peroxide for 10 min to block endogenous peroxidase activity, followed by incubation with 5% goat serum at 25°C for 30 min to prevent non‐specific binding. The sections were then incubated at 4°C for 16 h with primary antibodies against ZMIZ2 (HPA040716, 1:50; Sigma‐Aldrich) and DUX4 (#12790, 1:50; Santa Cruz Biotechnology, Dallas, TX, USA). After washing with PBS, the sections were incubated with a peroxidase‐conjugated goat anti‐rabbit secondary antibody for 30 min at 37°C. Immunostaining was visualized using diaminobenzidine tetrahydrochloride.

ZMIZ2 and DUX4 levels were evaluated using a semi‐quantitative immunoreactivity score (IRS), calculated as the product of staining intensity (graded as 0: negative, 1: weak, 2: moderate, and 3: strong) and percentage of positively stained tumor cells (graded as 0: 0%, 1: 1%–25%, 2: 26%–50%, 3: 51%–75%, and 4: 76%–100%). In the absence of an established cut‐off, the threshold for positivity was determined based on the IRS scores of 12 adjacent normal breast epithelial samples, the majority of which scored <4. Thus, IRS scores ≥4 and <4 were defined as positive and negative, respectively. To ensure interobserver reliability, all sections were independently scored by two pathologists blinded to the clinical data, with a high concordance rate (inter‐observer disagreement rate < 5%). Discrepant cases were resolved by consensus with a third pathologist.

### 5.4 Plasmids and Reagents

Lipofectamine 3000 (Invitrogen, Carlsbad, CA, USA) was used for transient transfection, and puromycin (#P9620; Sigma‐Aldrich) was used for selecting stably transfected cells. The plasmids, short hairpin RNAs (shRNAs), and sgRNAs packaged in lentiviral particles are listed in the Supplementary Methods [76]. Docetaxel (#HY‐B0011) and liquiritin (LIQ; #HY‐N0376) were purchased from MedChem Express (Shanghai, China).

### 5.5 Immunoblotting and Immunoprecipitation

Immunoblotting and IP assays were performed as previously described [21]. Briefly, total protein was extracted using lysis buffer (Pierce, Rockford, IL, USA) and quantified using the bicinchoninic acid method. Equal amounts of total protein (50 µg) were separated using 10% sodium dodecyl sulfate–polyacrylamide gel electrophoresis and transferred onto polyvinylidene fluoride membranes (Millipore, Billerica, MA, USA). The membranes were blocked and then incubated overnight at 4°C with the primary antibodies listed in the Supplementary Methods. After washing, the membranes were incubated with peroxidase‐conjugated anti‐mouse or anti‐rabbit IgG secondary antibodies (Proteintech, Wuhan, China) at 37°C for 2 h. Protein bands were visualized using enhanced chemiluminescence reagents (Pierce) and detected using a bio‐imaging system (DNR Bio‐Imaging Systems, Jerusalem, Israel).

### 5.6 Immunofluorescence Staining

The cells were fixed with 4% paraformaldehyde, permeabilized, and blocked with 3% bovine serum albumin. The cells were then incubated overnight at 4°C with primary antibodies against ZMIZ2 (1:100) and DUX4 (SAB5200019, 1:50; Sigma‐Aldrich), followed by incubation with tetramethyl rhodamine isothiocyanate (TRITC)‐ or fluorescein isothiocyanate (FITC)‐conjugated secondary antibodies at room temperature for 1 h. Nuclei were counterstained with 4′,6‐diamidino‐2‐phenylindole. Images were acquired using a Radiance 2000 laser scanning confocal microscope (Carl Zeiss, Oberkochen, Germany).

### 5.7 GST Pull‐Down Assay

The GST‐tagged DUX4 protein was expressed in *Escherichia coli* BL21 cells and purified using glutathione‐Sepharose affinity chromatography. Glutathione‐Sepharose beads (GE Healthcare, Waukesha, WI, USA) pre‐coupled with purified GST or GST‐DUX4 were incubated with lysates from HEK293 cells transfected with the HA‐ZMIZ2 plasmid overnight at 4°C. The bound complexes were then washed extensively and analyzed by immunoblotting.

### 5.8 RNA Extraction and RT‐qPCR

Total RNA was extracted and subjected to RT‐qPCR as previously described [21]. Gene expression levels were normalizednormalized to *GAPDH* mRNA levels. The primer sequences used are listed in Supplementary Table 1.

### 5.9 Chromatin immunoprecipitation (ChIP), ChIP‐seq, and ChIP‐qPCR

ChIP assays were performed using a commercial kit, according to the manufacturer's instructions (#17‐371; Millipore). For ChIP‐seq, MDA‐MB‐231 cells overexpressing DUX4 were cross‐linked with 1% formaldehyde at room temperature for 10 min, followed by quenching with 0.125 M glycine. The cells were then lysed by sonication. The chromatin lysates were incubated with an anti‐Flag antibody. For ChIP‐qPCR, four groups of MDA‐MB‐231 cells—NC, DUX4 overexpression alone, ZMIZ2 knockdown alone, and DUX4 overexpression combined with ZMIZ2 knockdown—were processed in parallel and incubated with an anti‐DUX4 antibody. Protein G agarose beads were added, and the mixtures were rotated at 4°C for 1 h, followed by overnight incubation with the appropriate antibodies at 4°C. After elution, the protein–DNA complexes were reversed. Immunoprecipitated DNA from the anti‐Flag ChIP was subjected to high‐throughput sequencing (Baihao Technology, Shenyang, China) for genome‐wide analysis. The immunoprecipitated DNA from the anti‐DUX4 ChIP was analyzed by quantitative PCR (qPCR) using specific primers for the LGR5 promoter region (forward, 5′‐CACGTCTTCACATGAGGAAGC‐3′; reverse, 5′‐TTGAATAGTGATATGGTGGAATG‐3′). The fold enrichment was calculated relative to the IgG control using the 2^–ΔΔCt method.

### 5.10 Dual‐luciferase Assay

Dual‐luciferase reporter assays were performed as previously described [21]. The luciferase reporter plasmids used in this study are described in detail in the Supplementary Methods.

### 5.11 Colony Formation, Migration, Matrigel Invasion, and MTS Assays

Cells stably transduced with the indicated plasmids were seeded in 6‐cm cell culture dishes (1,000 cells/dish) and incubated for 14 days. The colonies were washed with PBS, fixed, and stained with Giemsa solution. Colony numbers were counted manually. Protocols for the migration, Matrigel invasion, and MTS assays are detailed in the Supplementary Methods. All experiments were performed in three independent biological replicates.

### 5.12 Sphere Formation Assay

For the primary sphere formation assay, cells (1 × 10^3^) were seeded in 24‐well ultra‐low attachment plates (Costar, Cambridge, MA, USA) and cultured in serum‐free DMEM/F12 medium (Invitrogen) supplemented with 1× B27 (Invitrogen), 20 ng/mL epidermal growth factor (BD Biosciences, San Jose, CA, USA), and 4 µg/mL insulin (Sigma‐Aldrich) at 37°C in a humidified atmosphere containing 5% CO_2_ for 10–14 d. Mammospheres with a diameter >75 µm were counted in five randomly selected fields. For secondary sphere formation, primary mammospheres were collected by centrifugation at 800 *g*, dissociated into single cells using trypsin–EDTA, and filtered through a 70‐µm cell strainer. The resulting single cells were then reseeded at the same density in fresh ultra‐low attachment plates and cultured under identical conditions for an additional 10–14 d.

### 5.13 ALDEFLUOR Flow Cytometry

ALDEFLUOR assays were performed according to the manufacturer's instructions (STEMCELL Technologies, Vancouver, BC, Canada). Cell pellets were resuspended in 0.5 mL of ALDEFLUOR Assay Buffer and stored on ice or at 4°C. For each sample, an aliquot was incubated with ALDEFLUOR substrate in the presence or absence of the ALDH inhibitor diethylaminobenzaldehyde (DEAB) to serve as a negative control. Samples were analyzed using a CytoFlex flow cytometer (Beckman Coulter, Brea, CA, USA) equipped with a 488/513 nm channel for ALDEFLUOR or FITC detection. Data were analyzed using the FlowJo software (BD Biosciences).

### 5.14 Cell Apoptosis Assay

Cells were seeded in 6‐cm culture dishes and treated with docetaxel at the indicated concentrations for 48 h, followed by an additional 48 h of culture in drug‐free medium. The cells were collected and resuspended in 0.5 mL binding buffer containing 5 µL Annexin V–FITC and propidium iodide (PI) according to the manufacturer's instructions (GeneChem, Shanghai, China). Apoptosis was assessed by flow cytometry on an Accuri C6 Plus flow cytometer (BD Biosciences, Franklin Lakes, NJ, USA), and the data were analyzed using FlowJo software (v.10.0; BD Biosciences).

### 5.15 RNA Sequencing and Bioinformatic Analysis

Total RNA was extracted from MDA‐MB‐231, MDA‐MB‐231R, MDA‐MB‐468, MDA‐MB‐468R, and ZMIZ2‐overexpressing cells (Takara Bio, Kyoto, Japan), according to the manufacturer's instructions. RNA purity was determined using a NanoPhotometer (IMPLEN, Westlake Village, CA, USA). cDNA libraries were constructed from 1 µg total RNA using a PCR‐cDNA Sequencing Kit (#SQK‐PCS109; Nanopore Technologies, Oxford, UK), according to the manufacturer's protocol. Differentially expressed gene sets were identified using the R package DESeq2 (v.1.50.2) [77]. Genes with an adjusted *p*‐value (p.adj) < 0.05 and an absolute log_2_ fold change > 0.2 were considered statistically significant.

To prioritize clinically relevant candidates, the DEGs were cross‐referenced with three publicly available RNA‐seq datasets of docetaxel‐resistant TNBC from the GEO database: GSE162285 (docetaxel‐persistent MDA‐MB‐231 3D organoids vs. naïve MDA‐MB‐231 3D organoids), GSE21974 (breast cancer tissues before vs. after four cycles of neoadjuvant chemotherapy), and GSE250632 (MDA‐MB‐468 cells treated with docetaxel for 24 h vs. DMSO‐treated controls). These raw data were downloaded from the GEO database (https://www.ncbi.nlm.nih.gov/geo/) and using the same DESeq2 pipeline described above.

To interpret the biological implications of the DEGs, GO enrichment and KEGG pathway analyses were performed using the R package clusterProfiler (v.4.18.4) [78]. The GO terms were obtained from the GO database [79], and the pathway information was retrieved from the KEGG database [80]. All statistical plots and figures were generated using the R package ggplot2 (v.3.4.0) [81].

### 5.16 Liquid Chromatography (LC)–MS/MS

ZMIZ2‐interacting proteins were identified using MS. Gel pieces containing co‐immunoprecipitated ZMIZ2 were subjected to nano‐LC–MS/MS analysis on a Q Exactive mass spectrometer (Thermo Fisher Scientific) coupled with an Easy nLC system (Thermo Fisher Scientific). Raw MS/MS data were converted to MGF format using Proteome Discoverer (v.1.4; Thermo Fisher Scientific). Peptide identification was performed using Mascot software (v.2.3.01; Matrix Science, London, UK) against the UniProt database. A decoy database search was applied to estimate the false discovery rate, which was set at <0.05.

### 5.17 Virtual Screening and Molecular Docking

The crystal structure of ZMIZ2 was predicted using AlphaFold2 [82] and prepared using the Protein Preparation Wizard module in Schrödinger software (Schrödinger, New York, NY, USA), which included protein preprocessing, native ligand state regeneration, hydrogen‐bond assignment optimization, restrained energy minimization, and water molecule removal.

Ligands from 12 compound libraries were processed using the LigPrep module in Schrödinger to generate all possible 3D stereoisomers. ADME/T properties were evaluated using QikProp, with 51 parameters calculated and primarily assessed according to Lipinski's Rule of Five.

The active site of ZMIZ2 was identified using the SiteMap module, and an enclosing box was generated using the Receptor Grid Generation module to define the docking region. Molecular docking was performed using Glide with a hierarchical protocol: High‐Throughput Virtual Screening, followed by Standard Precision and Extra Precision docking. The best‐docked poses were further evaluated using Molecular mMechanics/generalized Born Surface Area binding free energy calculations. The compound with the lowest binding free energy was selected as the top candidate for subsequent experimental validation.

### 5.18 Surface Plasmon Resonance (SPR) Assay

The binding affinity of LIQ to ZMIZ2 was determined by SPR on a Biacore T200 instrument (GE Healthcare, Chicago, IL, USA). The sensor chip surface was activated by injecting a freshly prepared mixture of 400 mM EDC and 100 mM NHS (GE Healthcare) into the Fc1 and Fc4 channels at a flow rate of 10 µL/min for 800 s. ZMIZ2 (39 µg/mL) in 10 mM sodium acetate (NaAc, pH 4.0) was then immobilizedimmobilized onto the Fc4 channel at a flow rate of 10 µL/min for 950 s, reaching an immobilization level of ∼1408.3 RU. The remaining active sites were blocked by injecting 1 M ethanolamine hydrochloride (GE Healthcare) at a flow rate of 10 µL/min for 800 s. For binding kinetics, LIQ was serially diluted in running buffer [1× PBS‐P containing 5% dimethyl sulfoxide (DMSO); GE Healthcare] to concentrations of 0–10 000 µM. The diluted LIQ samples were injected over the Fc1–Fc4 channels at a flow rate of 30 µL/min for an association phase of 90 s, followed by a dissociation phase of 120 s. All binding experiments were performed in running buffer at 25°C.

### 5.19 Cellular Thermal Shift (CETSA) Assay

CETSA was performed to evaluate the binding of LIQ to ZMIZ2 in live cells. MDA‐MB‐231 cells were treated with either DMSO (control) or liquiritin (120 µM) for 4 h. Cells were then harvested, washed with PBS containing protease inhibitors, and heated at the indicated temperatures for 3 min in a metal bath. Following heat treatment, the cells were lysed in NP‐40 lysis buffer and subjected to three freeze–thaw cycles using liquid nitrogen. The lysates were clarified by centrifugation at 20 000 × *g* for 20 min at 4°C, and the resulting soluble fractions were collected. The supernatants were mixed with the loading buffer, boiled, and analyzed by immunoblotting.

### 5.20 Animal Experiments

All animal experiments were approved by the Experimental Animal Ethics Committee of China Medical University (Approval No.: KT20250561) and conducted in accordance with the National Institutes of Health (NIH) Guidelines for the Care and Use of Laboratory Animals (NIH Publication No. 8023, revised 1978). Female BALB/c nude mice (5–6 weeks old, 16–17 g) were purchased from Shanghai Model Organisms CentreCenter (Shanghai, China) and housed in a specific pathogen‐free facility under a controlled environment (22 ± 2°C, 55 ± 5% humidity) with a 12‐h light/dark cycle. Mice were provided with standard chow and water ad libitum and acclimatized for one week prior to the experiment.

For subcutaneous tumor models, mice weighing 16.0–17.0 g were randomly assigned into three groups (*n* = 5 per group): vehicle control, docetaxel alone, and docetaxel combined with LIQ using a computer‐generated randomization schedule. The investigators performing the tumor measurements were blinded to the allocation sequence. When the tumor volume reached ∼50 mm^3^, treatment (docetaxel: 10 mg/kg, LIQ: 20 mg/kg) was initiated and administered three times per week for four consecutive weeks via intraperitoneal injection. To evaluate systemic toxicity, body weights were recorded once a week throughout the experimental period. The investigators performing tumor volume/weight measurements and data collection were blinded to the group allocation. At the end of the study, all mice were euthanized by CO_2_ asphyxiation, and tissues were collected for further analysis.

For limiting dilution xenograft assays, 8‐week‐old female nude mice were randomly assigned to experimental groups (*n* = 6 per group) using a computer‐generated randomization schedule [83]. MDA‐MB‐231 cells overexpressing ZMIZ2, alone or in combination with DUX4 and LGR5 knockdown, as well as MDA‐MB‐231R cells treated with LIQ or docetaxel, were suspended at varying densities in a 1:1 mixture of DMEM and Matrigel (BD Biosciences) and injected subcutaneously into the mice at 3 × 10^4^, 1 × 10^4^, 3 × 10^3^, or 1 × 10^3^ cells/mouse. Tumor growth was monitored, and data were collected by investigators blinded to the group allocation. Tumor incidence was analyzed by limiting dilution analysis using ELDA software (http://bioinf.wehi.edu.au/software/elda/) [84].

For orthotopic mammary fat pad injection models, MDA‐MB‐231R cells expressing control sgRNA, sgZMIZ2‐2, or sgZMIZ2‐2 combined with ZMIZ2 re‐expression were randomly assigned for injection into the mammary fat pads of female nude mice (*n* = 5 per group) using a computer‐generated randomization schedule.

For the drug‐treated cohort, docetaxel (10 mg/kg) was administered three times per week for 4 weeks, starting 1 week after injection, and the remaining mice were monitored without docetaxel treatment. MDA‐MB‐231 cells expressing control vectors, ZMIZ2, DUX4, or sgDUX4 combined with ZMIZ2 were injected into the mammary fat pads (*n* = 5 per group) and allowed to grow for 5 weeks. In a separate cohort, MDA‐MB‐231R cells bearing the same genetic modifications were injected (*n* = 5 per group) and treated with docetaxel (10 mg/kg) starting at 1 week post‐injection. Tumor volume was measured weekly using calipers and calculated as follows: *V* = 0.5 × (length × width^2^). Tumor weight was measured at the endpoint by investigators blinded to the group allocation.

### 5.21 Statistical Analysis

All statistical analyses were performed using GraphPad Prism software (version 8.0; GraphPad Software, San Diego, CA, USA). Differences between two groups were compared using the unpaired Student's *t*‐test, whereas comparisons among multiple groups were performed using one‐way or two‐way ANOVA followed by Tukey's, Šídák's, or Dunnett's multiple comparisons test, as appropriate. *P* < 0.05 was considered statistically significant. For specific panels, the corresponding statistical methods are detailed in the figure legends.

## Author Contributions

**Xueting Gan**, **Shulei Sun**, and **Ran Hu** contributed equally to this work. **Xueting Gan**: Writing – original draft, Methodology, Validation, Formal analysis, Conceptualization, Software, Investigation, Visualization, Writing – review & editing. **Shulei Sun**: Data curation, Methodology, Formal analysis, Conceptualization, Investigation, Validation. **Ran Hu**: Methodology, Formal analysis, Data curation, Conceptualization, Validation, Investigation. **Yuheng Feng**: Methodology, Formal analysis, Investigation. **Xinhao Wu**: Methodology, Formal analysis, Investigation. **Fangqi Liu**: Methodology, Formal analysis, Investigation. **Xuyong Lin**: Writing – review & editing, Funding acquisition. **Xiupeng Zhang**: Resources, Software, Writing – review & editing. **Xuezhu Rong**: Writing – review & editing, Supervision, Project administration, Funding acquisition, Conceptualization. **Qiang Han**: Conceptualization, Funding acquisition, Writing – review & editing, Writing – original draft, Supervision, Project administration.

## Funding

This work was supported by the National Natural Science Foundation of China (grants 81902986 to Qiang Han, grants 82003119 to Xuezhu Rong), and the Natural Science Foundation of Liaoning Province (grants 2022‐MS‐198 to Xuyong Lin).

## Ethics Approval and Consent to Participate

All patients provided informed consent, and the study was approved by the Ethics Committee of China Medical University (No. LS [2025]003). All animal subjects were treated according to the National Institutes of Health Guidelines for the Care and Use of Laboratory Animals (NIH Publication No. 8023, revised 1978). The animal experiments were approved by the experimental animal ethics guidelines issued by the China Medical University (No. KT20250561).

## Conflicts of Interest

The authors declare that they have no conflicts of interest.

## Supporting information




**Supporting File 1**: advs78319‐sup‐0001‐SuppMat.docx.

**Supporting File 2**: advs78319‐sup‐0002‐TableS2.xlsx.

## Data Availability

The raw RNA‐seq data generated in this study have been deposited in the Sequence Read Archive (SRA) database under the BioProject ID PRJNA1493283. The processed sequencing data, including gene expression matrices and differentially expressed gene lists, are available within the article and its supplementary information files. All other data are available from the corresponding author upon reasonable request.
