# Supplementary material for: ZMIZ2–DUX4–LGR5 Signaling Axis Maintains Stemness Properties and Facilitates Docetaxel Resistance in Triple‐Negative Breast Cancer
Source: Adv Sci (Weinh). 2026 Oct 22:e78319. Online ahead of print. doi: 10.1002/advs.78319 (PMC13691226; doi:10.1002/advs.78319)
Supplement: Supplementary file 1 [file ADVS-9999-e78319-s001.docx]

Supporting Information

**ZMIZ2–DUX4–LGR5 Signaling Axis Maintains Stemness Properties and Facilitates Docetaxel Resistance in Triple-negative Breast Cancer**

*Xueting Gan^1,2^****^#^****, Shulei Sun^3^****^#^****, Ran Hu^4^****^#^****, Yuheng Feng^1^, Xinhao Wu^5^, Fangqi Liu^1^,*

*and Xuyong Lin^1^*, Xiupeng Zhang^1^*, Xuezhu Rong^6^*, Qiang Han^1^**

**Supplementary Table**

Table 1. The primers used for RT-qPCR are listed below:

|  | Primer sequences (5′→3′) |
| --- | --- |
| *ZMIZ2* | *5'ATGAACCCTACTGGCATAGGAG3'*  *5'GCCCAGGATACCCATGTTGG'3* |
| *GAPDH* | *5' GCCATCAATGACCCCTTCATTGAC '3*  *5' TCGCTCCTGGAAGATGGTGATG '3* |
| *LGR5* | *5'GAGGATCTGGTGAGCCTGAGAA'3*  *5'CATAAGTGATGCTGGAGCTGGTAA'3* |
| *CD133* | *5'CCTCCCTGTTGGTGATTTGTAT'3*  *5'CCTTGTCCTTGGTAGTGTTGT'3* |
| *CD44* | *5'GCATTGCAGTCAACAGTCGAAGA'3*  *5'CCTTGTTCACCAAATGCACCA'3* |

**Supplementary Figures**

**Figure S1.**

**Figure S1. Identification and clinical relevance of ZMIZ2 as a novel mediator of docetaxel resistance in TNBC**

**(A–B)** Colony formation assays evaluating the clonogenic survival of resistant and parental cells following vehicle or docetaxel treatment (n = 3 independent experiments). **(C)** Heatmap illustrating the differentially expressed genes (DEGs) between docetaxel-resistant cells and their matched parental counterparts. **(D–E)** Volcano plots showing the differentially expressed genes between the resistant sublines and parental cells.For panels A–B, data are presented as the mean ± standard deviation (SD) from three biologically independent samples. Statistical significance was determined using two-way ANOVA followed by Šídák's multiple comparisons test. *P*-values are indicated in the corresponding panels.

**Figure S2.**

**Figure S2. ZMIZ2 enhances the malignant phenotype and confers drug resistance in TNBC.**

**(A)** Immunoblotting analysis confirming the efficiency of ZMIZ2 knockdown in the indicated TNBC cell lines following lentiviral transduction. **(B–H)** In *vitro* functional assays showing that ZMIZ2 knockdown significantly suppressed proliferation of both docetaxel-resistant and parental TNBC cells (B–C), as well as colony formation (D–E) and migration/invasion (F–G) of parental TNBC cells. Conversely, ZMIZ2 overexpression significantly promoted proliferation of MDA-MB-231 and MDA-MB-468 cells (H) (n = 3 independent experiments). Scale bar: 50 μm; magnification: 200×. **(I)** Schematic diagram of the orthotopic tumorigenesis model. Representative images of mouse orthotopic tumors are shown for mice receiving control sgRNA, sgRNA-ZMIZ2, or sgRNA-ZMIZ2 with ZMIZ2 re-expression, with or without docetaxel treatment. **(J–K)** Tumor volume (J) was measured weekly throughout the indicated period, and tumor weight (K) was recorded at the endpoint (n = 5 mice per group). For panels B–H, data are presented as the mean ± SD from three biologically independent samples. Statistical significance was determined using two-way ANOVA followed by Dunnett's (B–C) or Šídák's multiple comparisons test (H), one-way ANOVA followed by Dunnett's multiple comparisons test (D–G).

For panels J–K, data are presented as the mean ± SD from five mice per group. Statistical significance was determined using two-way (J) or one-way (K) ANOVA followed by Tukey's multiple comparisons test. *P*-values are indicated in the corresponding panels.

**Figure S3.**

**Figure S3. ZMIZ2 functions as a transcriptional co-activator of DUX4 via its MIZ domain.**

**(A–B)** The HDOCK-predicted binding mode (http://hdock.phys.hust.edu.cn/) (A) and detailed interface residues (B­­) were visualized using PyMOL. **(C)** PRODIGY-predicted binding parameters for the HDOCK Model_1. The table summarizes the binding affinity (ΔG), dissociation constant (K_d_), total intermolecular contacts, contact type distribution, and interface residue composition of the top-ranked docking model.

**Figure S4.**

**Figure S4. ZMIZ2 cooperates with BRG1 and BAF57 to facilitate DUX4-mediated LGR5 transcription.**

(A) Schematic diagram of the wild-type and DUX4 binding site core-base-substituted LGR5 promoter luciferase reporter constructs.

(B) Dual-luciferase reporter assays showing the synergistic activation of the wild-type LGR5 promoter by ZMIZ2 and DUX4, whereas this effect was abolished in the DUX4 binding site core-base-substituted mutant. Renilla luciferase was used for normalization.

**(C)** Co-IP assays confirming the formation of the ZMIZ2–DUX4–BRG1–BAF57 complex. HEK293 cells were co-transfected with HA-ZMIZ2, Flag-DUX4, and His-BRG1 or GFP-BAF57, either alone or in combination, and subjected to immunoprecipitation with anti-HA.

**Figure S5.**

**Figure S5. The ZMIZ2–DUX4–LGR5 axis enhances stemness-associated properties and tumorigenicity in TNBC.**

**(A–B)** RT-qPCR analysis of CD133 and CD44 mRNA expression in MDA-MB-468 cells co-transduced with ZMIZ2 and DUX4 or with ZMIZ2 and sgDUX4 (n = 3 independent experiments). **(C–D)** Spheroid formation assays showing that co-transduction of ZMIZ2 and DUX4 enhanced sphere-forming capacity (C), whereas DUX4 knockdown reversed this effect (D) (n = 3 independent experiments). Scale bar: 100 μm; magnification,: 100×. **(E–F)** ALDEFLUOR flow cytometry assays showing that ZMIZ2 and DUX4 co-overexpression significantly increased the percentage of ALDH+ cells (E), and this effect was reversed upon DUX4 knockdown (F) (n = 3 independent experiments).

For panels A–F, data are presented as the mean ± SD from three biologically independent samples. Statistical significance was determined using one-way ANOVA followed by Dunnett's multiple comparisons test (A–F). *P*-values are indicated in the corresponding panels.

**Figure S6.**

**Figure S6. ZMIZ2 promotes drug resistance and the malignant phenotype in TNBC via DUX4.**

**(A–B)** MTS assays showing the dose-response curves of MDA-MB-468 cells co-transduced with ZMIZ2 and DUX4 or with ZMIZ2 and DUX4 sgRNA after 48 h of docetaxel treatment (8, 16, 32, 64, 128, and 256 µM). Overexpression of both ZMIZ2 and DUX4 shifted the dose-response curves rightward and significantly elevated the IC₅₀ values compared with control cells (NC)(A). Conversely, DUX4 knockdown shifted the dose-response curves leftward and significantly reduced the IC₅₀ value in ZMIZ2-overexpressing cells (B) (n = 3 independent experiments). **(C–D)** Flow cytometry analysis of apoptosis rates in MDA-MB-468 cells co-transduced with ZMIZ2 and DUX4 sgRNA under DMSO or docetaxel treatment (n = 3 independent experiments). **(E–J)** In vitro functional assays showing that co-overexpression of ZMIZ2 and DUX4 significantly enhanced colony formation (E), migration, and invasion (G–H) in MDA-MB-468 cells. Conversely, DUX4 knockdown abolished these ZMIZ2-mediated effects (F, I–J) (n = 3 independent experiments). Scale bar: 50 μm; magnification: 200×. For panels A–J, data are presented as the mean ± SD from three biologically independent samples. Statistical significance was determined using two-way ANOVA followed by Šídák's multiple comparisons test (C–D) or one-way ANOVA followed by Dunnett's multiple comparisons test (A–B, E–J). *P*-values are indicated in the corresponding panels.

**Figure S7.**

**Figure S7. ZMIZ2 promotes drug resistance and the malignant phenotype in TNBC via DUX4.**

**(A)** Immunoblotting analysis confirming the efficiency of ZMIZ2 or DUX4 overexpression, and DUX4 knockdown, in the indicated TNBC cell lines following lentiviral transduction.

**(B–E)** In vitro functional assays showing that overexpression of ZMIZ2 alone or DUX4 alone significantly enhanced colony formation (B–C), migration, and invasion (D–E) of MDA-MB-231R and MDA-MB-468R cells. Conversely, DUX4 knockdown abolished these ZMIZ2-mediated effects (n = 3 independent experiments). Scale bar: 50 μm; magnification: 200×.

For panels B–E, data are presented as the mean ± SD from three biologically independent samples. Statistical significance was determined using one-way ANOVA followed by Dunnett's multiple comparisons test. *P*-values are indicated in the corresponding panels.

**Figure S8.**

**Figure S8. Liquiritin sensitizes TNBC to docetaxel and suppresses stemness-associated properties by targeting ZMIZ2.**

**(A)** Immunoblotting analysis of ZMIZ2 and DUX4 protein levels in the indicated cell lines treated with or without LIQ (MDA-MB-231R: 40 µM; MDA-MB-468R: 20 µM). **(B)** Drug sensitivity of LIQ in ZMIZ2‐knockdown MDA‐MB‐468R cells. IC₅₀ values were estimated using a non-linear fitting method. **(C–H)** In *vitro* functional assays showing the biological effects of LIQ on docetaxel-resistant MDA-MB-231R and MDA-MB-468R cells under ZMIZ2 knockdown conditions, as assessed by colony formation (C), Transwell migration and invasion (D–E; scale bar: 50 µm; magnification: 200×), spheroid formation (F; scale bar: 100 µm; magnification: 100×), and ALDEFLUOR flow cytometry assays (G–H) (n = 3 independent experiments). For panels **C–G**, data are presented as the mean ± SD from three biologically independent samples. Statistical significance was determined using two-way ANOVA followed by Tukey's multiple comparisons test. *P-*values are indicated in the corresponding panels.

**Figure S9**

**Figure S9. Liquiritin sensitizes TNBC to docetaxel and suppresses stemness-associated properties by targeting ZMIZ2.**

**(A–B)** Effects of LIQ combined with docetaxel on drug-resistant MDA-MB-468R cells, as determined by drug susceptibility (A) and apoptosis assays (B). Synergy was calculated using Compusyn software (CI < 1.0). **(C–D)** Colony formation (C) and Transwell (D) assays showing the synergistic inhibitory effects of LIQ and docetaxel on colony formation, migration, and invasion in MDA-MB-468R cells (n = 3 independent experiments). Scale bar: 50 μm; magnification: 200×. **(E–F)** Schematic diagram of the subcutaneous tumorigenesis model (E) and body weight changes (F) following 4 weeks of drug treatment (docetaxel: 10 mg/kg; LIQ: 20 mg/kg). **(G–H)** Spheroid formation (E) and ALDEFLUOR flow cytometry (F) assays showing that LIQ and docetaxel synergistically inhibited sphere formation and reduced the proportion of ALDH+ cells in MDA-MB-468R cells (n = 3 independent experiments). Scale bar: 100 μm; magnification: 100×.

For panels B–H, data are presented as the mean ± SD from three biologically independent samples. Statistical significance was determined using one-way ANOVA followed by Tukey's (B) or Dunnett's multiple comparisons test (C–D, G–H). For panel F, data are presented as the mean ± SD from five mice per group. Statistical significance was determined using two-way ANOVA, followed by Dunnett's multiple comparisons test. *P*-values are indicated in the corresponding panels.

**Supplementary Methods**

**1 | Antibodies**

ZMIZ2 (#A17301, ABclonal, Wuhan, China; #HPA040716, Sigma-Aldrich), Flag-tag (#14793, Cell Signaling Technology, Danvers, MA, USA), GST-tag (#2625, Cell Signaling Technology), His-Tag (#2365, Cell Signaling Technology, Danvers, MA, USA), GFP(#2555, Cell Signaling Technology, Danvers, MA, USA), HA-tag (#sc-7392, Santa Cruz), DUX4 (#12790, Santa Cruz), LGR5 (#ab273092, Abcam, Cambridge, UK), BRG1 (#ab110641, Abcam), BAF57 (#ab131328, Abcam) and GAPDH (#sc-365062, Santa Cruz).

**2 | Plasmids, short-hairpin RNAs (shRNAs) and sing-guide RNAs (sgRNAs)**

pCMV-CMV-mCherry-MCS-3×HA,pCMV-CMV-mCherry-ZMIZ2-3×HA,pCMV-CMV-mCherry-ZMIZ2-Del(554-570aa)-3×HA,pCMV-CMV-mCherry-ZMIZ2-(1-694aa)-3×HA, pCMV-CMV-mCherry-ZMIZ2-(1-590aa)-3×HA;control-sgRNA(GCACTACCAGAGCTAA CTTCA) and pLentiCRISPRV2-U6-ZMIZ2-sgRNA-cas9(#1:GCAAGGCGTGTACAGCCG CG, #2: CAGTAGGGTTCATGCCAGCG); pLentiCRISPRV2-U6-DUX4-sgRNA-cas9 (#1: CTTCGCCCACACCGGCGCCGT, #2: GACGGCGACGGAGACTCGTT) were constructed and purchased from MiaoLingBio (Wuhan, China). pCMV6 empty vector (#PS-100001) and Myc/DDK-pCMV6-DUX4 (#RC238145) were purchased from Origene (Rockville, MD, USA). shBAF57(#sc-45940-SH), shBrg1(#sc-44287-SH) and LGR5 CRISPR/Cas9 KO Plasmid (#sc-400726) were purchased from Santa Cruz Biotechnology. The Flag-BAF57 plasmid was constructed using primers described previously [76]. The GFP-BAF57 plasmid was constructed using primers designed in this study: forward, 5'-CGCGGGATCCATGTCAAAAAGACCATCTTATGCC'-3; reverse, 5′- CCGCCTCGAGTTATTCTTTTTTCTCATCTTCTGGTATG -3′. The His-BRG1 plasmid was constructed using primers designed in this study: forward, 5′- TCCAGCACAGTGGCGGCCGCATGTCCACTCCAGACCCACCC -3′; reverse, 5′- AACGGGCCCTCTAGACTCGATCAGTCTTCTTCGCTGCCACTTCCTG -3′.

**3 | Dual-luciferase assay**

LGR5 transcriptional activity was measured using a dual-luciferase reporter assay. The pGL3-Basic and, pGL3-Basic-LGR5 promoter wild-type, and mutant constructs were generated by GenePharma (Shanghai, China). Cells were co-transduced with the indicated expression plasmids and a Renilla luciferase reporter for signal normalization. Dual-luciferase assays were performed according to the manufacturer’s protocol (Promega, Madison, WI, USA). Data were normalized to the empty vector control and are presented as the mean ± SD

**4 | Migration, Matrigel invasion and MTS assays**

For Transwell assays, cell migration and invasion were assessed using 24-well Transwell chambers (8-μm pore size; Costar, Corning, NY, USA). For invasion assays, the upper surface of the inserts was pre-coated with 20 μL of Matrigel (1:5 dilution; BD Biosciences, Franklin Lakes, NJ, USA). At 48 h post-transduced, cells were trypsinized and resuspended in 200 μL of serum-free medium at a density of 5 × 10⁴ cells per well and seeded into the upper chamber. Medium containing 20% fetal bovine serum (FBS) was added to the lower chamber as a chemoattractant. After 16 h of incubation, the cells that had migrated or invaded through the membrane were fixed, stained, and counted in five randomly selected high-power fields under a microscope.

For the MTS assay, cell viability was measured using a 3-(4,5-dimethylthiazol-2-yl)-5-(3-carboxymethoxyphenyl)-2-(4-sulfophenyl)-2H-tetrazolium (MTS) kit (Promega, Madison, WI, USA). At 48 h post-transduction, cells were seeded into 96-well plates at a density of 3 × 10³ cells per well in medium containing 10% FBS. After the indicated incubation period, 20 μL of MTS solution (2 mg/mL) was added to each well and incubated at 37 °C for 2 h. The absorbance was measured using a spectrophotometer.
